# Rewiring *Escherichia coli* to transform formate into methyl groups

**DOI:** 10.1186/s12934-025-02674-4

**Published:** 2025-03-07

**Authors:** Michael K. F. Mohr, Ari Satanowski, Steffen N. Lindner, Tobias J. Erb, Jennifer N. Andexer

**Affiliations:** 1 Institute of Pharmaceutical Sciences, University of Freiburg, Albertstr. 25, 79104 Freiburg, Germany; 2 Max Planck Institute for Terrestrial Microbiology, Karl-Von-Frisch-Straße 10, 35043 Marburg, Germany; 3 Max Planck Institute of Molecular Plant Physiology, Am Mühlenberg 1, 14476 Potsdam-Golm, Germany; 4 Department of Biochemistry, Charité Universitätsmedizin Berlin, Corporate Member of Freie Universität Berlin and Humboldt-Universität, Charitéplatz 1, 10117 Berlin, Germany; 5 LOEWE Center for Synthetic Microbiology (SYNMIKRO), Philipps University of Marburg, Marburg, Germany

**Keywords:** Formic acid, Methylation, SAM, C_1_-building block, Methyltransferase, Biotransformation

## Abstract

**Background:**

Biotechnological applications are steadily growing and have become an important tool to reinvent the synthesis of chemicals and pharmaceuticals for lower dependence on fossil resources. In order to sustain this progression, new feedstocks for biotechnological hosts have to be explored. One-carbon (C_1_-)compounds, including formate, derived from CO_2_ or organic waste are accessible in large quantities with renewable energy, making them promising candidates. Previous studies showed that introducing the formate assimilation machinery from *Methylorubrum extorquens* into *Escherichia coli* allows assimilation of formate through the C_1_-tetrahydrofolate (C_1_-H_4_F) metabolism. Applying this route for formate assimilation, we here investigated utilisation of formate for the synthesis of value-added building blocks in *E. coli* using *S*-adenosylmethionine (SAM)-dependent methyltransferases (MT).

**Results:**

We first used a two-vector system to link formate assimilation and SAM-dependent methylation with three different MTs in *E. coli* BL21. By feeding isotopically labelled formate, methylated products with 51–81% ^13^C-labelling could be obtained without substantial changes in conversion rates. Focussing on improvement of product formation with one MT, we analysed the engineered C_1_-auxotrophic *E. coli* strain C_1_S. Screening of different formate concentrations allowed doubling of the conversion rate in comparison to the not formate-supplemented BL21 strain with a share of more than 70% formate-derived methyl groups.

**Conclusions:**

Within this study transformation of formate into methyl groups is demonstrated in *E. coli*. Our findings support that feeding formate can improve the availability of usable C_1_-compounds and, as a result, increase whole-cell methylation with engineered *E. coli*. Using this as a starting point, the introduction of additional auxiliary enzymes and ideas to make the system more energy-efficient are discussed for future applications.

**Graphical abstract:**

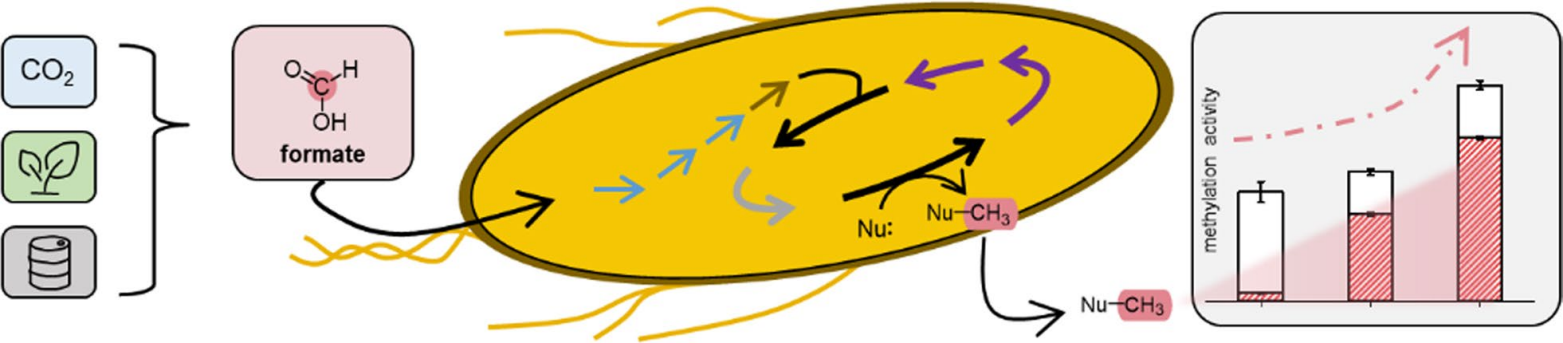

**Supplementary Information:**

The online version contains supplementary material available at 10.1186/s12934-025-02674-4.

## Background

The majority of industrial processes depends on fossil resources for the synthesis of compounds such as natural products, fuels, polymers, or fine chemicals including pharmaceuticals [1, 2]. With depleting fossil resources and the emerging effects of climate change, defossilisation is an important strategy to create a greener and more sustainable chemical industry [1]. Development of biochemical applications utilising alternative and renewable feedstocks to support or replace existing chemical processes is key to support this transformation [3,4]. One promising approach is the application of CO_2_-derived feedstocks in chemobiohybrid processes, *i.e.* initial (electro)chemical reduction of the greenhouse gas to produce reduced one-carbon (C_1_-)compounds such as formate and their subsequent valorisation through biotechnological efforts [5–7]. Additionally, enzymatic strategies for the less energy-intensive reduction of CO_2_ are investigated which will allow utilisation of CO_2_-derived formate for one-step whole-cell biocatalysis without prior electrochemical reduction in future applications [8, 9]. Formate has the benefit of stability, biodegradability, ease of handling and low toxicity, which means that it poses a low risk towards humans and the environment and is in accordance with many principles of green chemistry [7, 10–12]. For this reason, the development of biotechnological strategies based on the C_1_-compound formate is an emerging field [7, 10, 13].

**Fig. 1:**
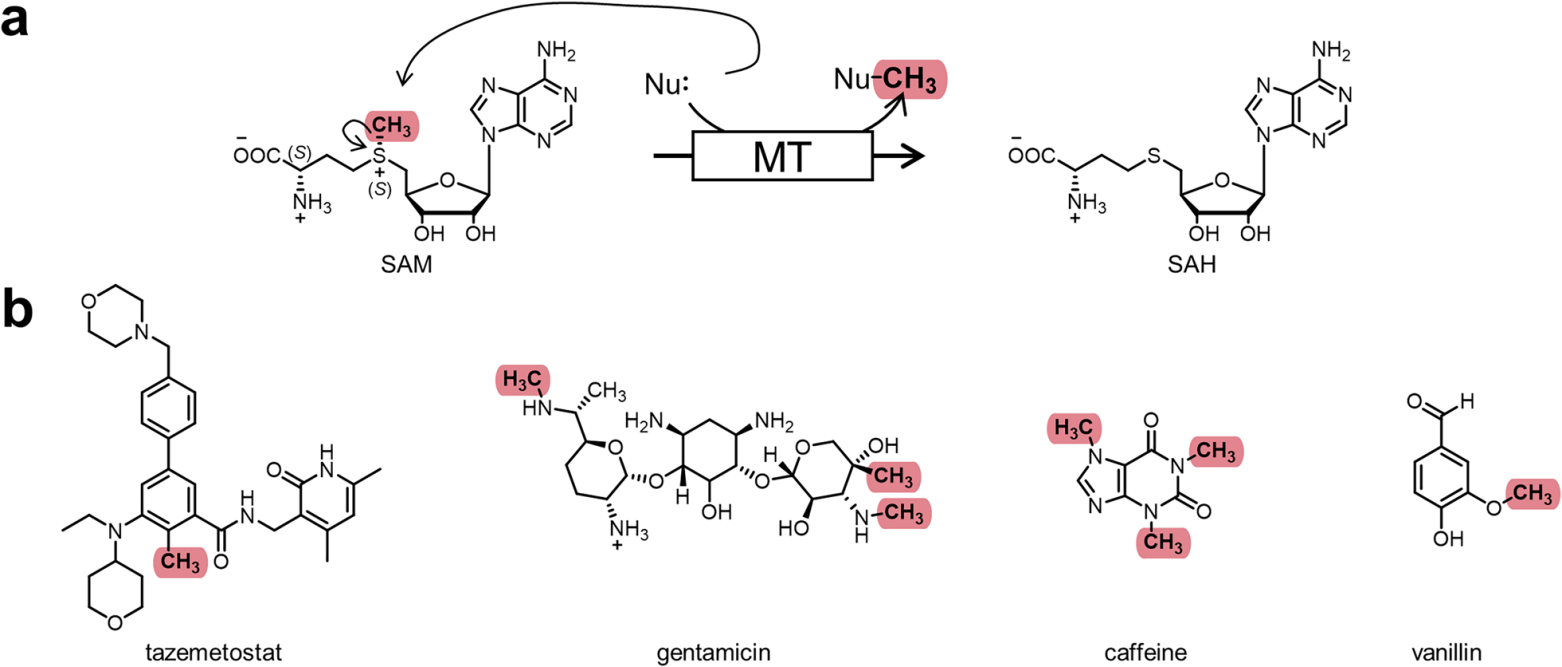
SAM-dependent methylation. **a** Reaction mechanism of *S*-adenosylmethionine (SAM)-dependent transmethylation of a nucleophilic substrate by methyltransferases (MTs) creating the byproduct *S*-adenosylhomocysteine (SAH). **b** Pharmaceuticals and natural compounds carrying “magic methyl” groups, which significantly influence potency. The relevant methyl in tazemetostat was chemically introduced during lead optimisation, *C-*methylation of gentamicin is mediated by a radical SAM MT, other methyl groups are introduced by conventional MTs

In nature, one important strategy to transfer C_1_-compounds onto various molecules is *S*-adenosylmethionine (SAM)-dependent methylation catalysed by methyltransferases (MTs, Fig. 1a) [14–16]. SAM-dependent methylation is involved in important processes such as DNA or histone methylation for epigenetic regulation of gene expression [17, 18]. Various natural products with biological activity are methylated and methylation of small molecular drugs at specific positions has been shown to increase their potency by up to three orders of magnitude, called the “magic methyl effect” [19]. This makes selective methylation an important step in lead structure optimisation during development of new pharmaceuticals (Fig. 1b). Chemical introduction of methyl groups is in many cases challenging to direct and commonly mediated under energy-consuming conditions by the use of toxic and environmentally harmful methylation agents such as methyl iodide or diazomethane [20]. In comparison to chemical methylation, MTs introduce the methyl group stereo-, regio- and chemoselectively, only synthesising one specific product; by including radical SAM MTs, chemically inaccessible sp^3^-hybridised carbons could be targeted as well [14, 21]. While chemical methylation is not the major driving force for the use of fossil resources, it is a hazardous technique necessary for countless syntheses, and would benefit from substitution by a strategy which is safer, more environmental-friendly and, hence, more in accordance with the principles of green chemistry; an example for such a substitution strategy is enzymatic, SAM-dependent methylation [12, 15, 16, 22, 23].

**Fig. 2:**
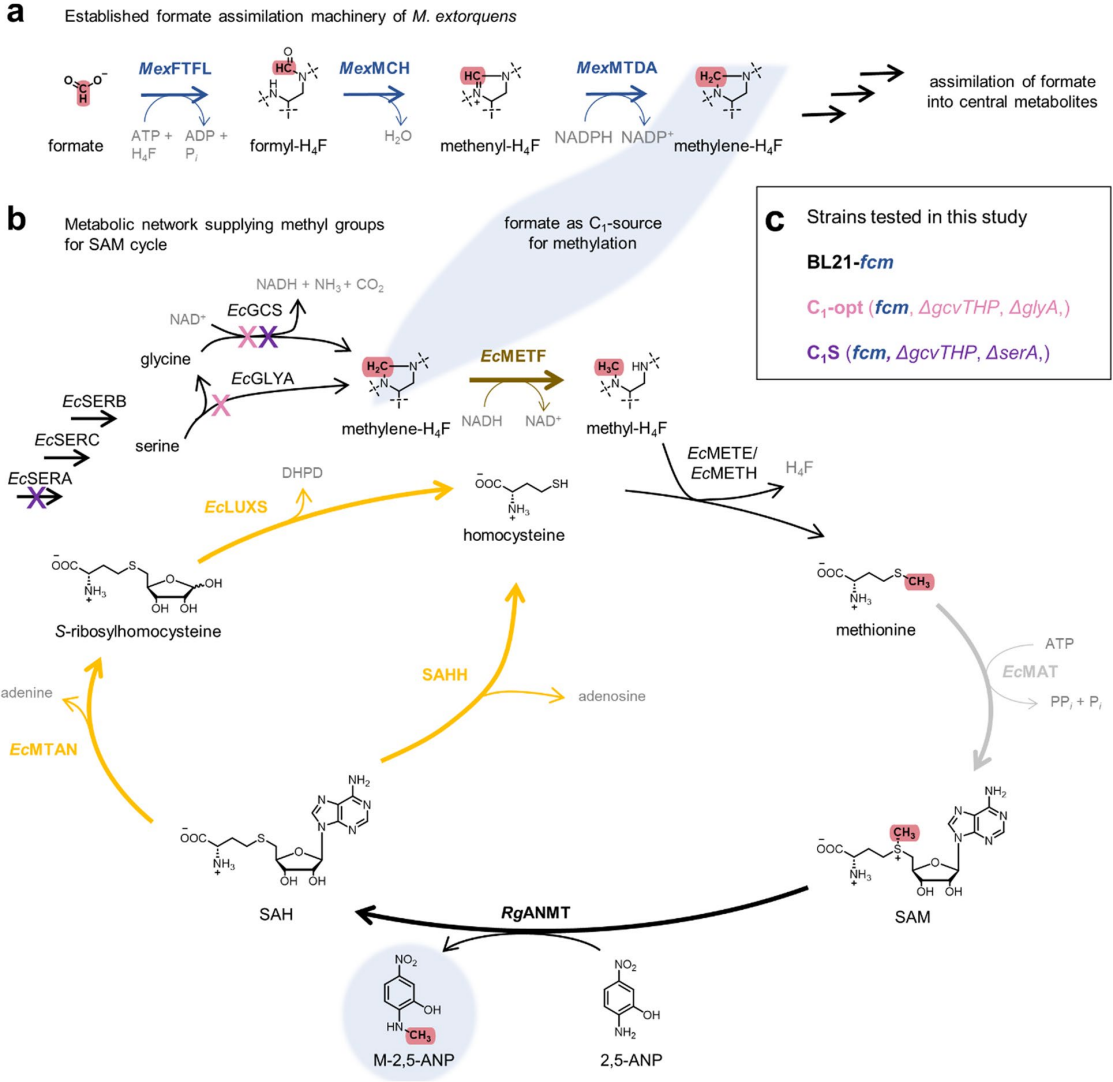
Metabolic network to convert formate into transferable methyl groups. **a** Established formate assimilation machinery consisting of *Mex*FTFL, *Mex*MCH and *Mex*MTDA from *M. extorquens* (together abbreviated as “FCM”) as it is implemented for the generation of synthetic formatotrophic *E. coli*. **b** Metabolic pathway for the native generation of C_1_-H_4_F metabolites from serine by *Ec*GLYA and glycine by *Ec*GCS for the supply of methyl groups to the SAM regeneration cycle. Heterologous introduction of an MT and supply of the MT substrate, here *Rg*ANMT with 2,5-ANP, enable formation of the methylated product and the byproduct SAH, which can be degraded to homocysteine either by *Ec*MTAN and *Ec*LUXS, or by heterologously introduced SAHH. The connection between formate assimilation and C_1_-H_4_F supply for the SAM cycle via methylene-H_4_F is highlighted in light blue; genes deleted in this study are labelled in the corresponding colour of the strains shown in (**c**). *2,5-ANP* - 2,5-aminonitrophenol, *ATP* - adenosine-5´-triphosphate, *ADP* - adenosine-5´-diphosphate, *C*_*1*_ - one carbon, *DHPD* - 4,5-dihydroxy-2,3-pentanedione, *H*_*4*_*F* - tetrahydrofolate, *NAD(P)H* - nicotinamide adenine dinucleotide (phosphate), *M-2,5-ANP - N*-methyl-2,5-aminonitrophenol, *SAM - S*-adenosylmethionine, *SAH - S*-adenosylhomocysteine, *EcGCS* - glycine cleavage system from *E. coli*, *EcGLYA* - serine hydroxymethyltransferase from *E. coli*, *EcLUXS - S*-ribosylhomocysteine lyase from *E. coli*, *EcMAT -* methionine adenosyltransferase from *E. coli*, *EcMETE/EcMETH -* methionine synthase from *E. coli*, *EcMETF -* methylene-H_4_F reductase from *E. coli*, *EcMTAN - S*-methyl-5´-thioadenosine/SAH nucleosidase from *E. coli*, *EcSERA -* 3-phospho-glycerate dehydrogenase from *E. coli*, *EcSERB -* phosphoserine phosphatase from *E. coli*, *EcSERC -* phosphoserine aminotransferase from *E. coli*, *MexFTFL -* formate-H_4_F ligase from *M. extorquens*, *MexMCH -* formyl-H_4_F cyclohydrolase from *M. extorquens*, *MexMTDA -* methylene-H_4_F dehydrogenase A from *M. extorquens*, *RgANMT -* anthranilate *N*-MT from *Ruta graveolens*

Intracellular methylation activity is often a major limiting factor when natural compounds are produced in biotechnological hosts, emphasising the need to develop strategies to improve conversion rates to synthesise methylated products [22–25]. Optimisation of intracellular SAM availability and acceleration of the cofactor’s regeneration can improve the formation of methylated products using whole-cell methylation [22–26]. One strategy which proved successful is increasing concentrations of the SAM precursor methionine, in particular its regeneration from homocysteine [23, 26–28]. In *E. coli*, homocysteine is methylated to methionine through transfer of a methyl group from methyl-tetrahydrofolate (methyl-H_4_F) catalysed by one of the methionine synthases *Ec*METE and *Ec*METH (Fig. 2) [29]. Majorly, the C_1_-H_4_F pool is fuelled from serine and glycine, catalysed by serine hydroxymethyltransferase (*Ec*GLYA) and the glycine cleavage system (*Ec*GCS), respectively [30, 31]. The C_1_-H_4_F pool supplies C_1_-carbon units for the synthesis of nucleobases and amino acids such as methionine or histidine, as well as for formylation of methionine, making it essential for protein production and cell growth [6, 32, 33]. *Okano et al.* reported that increasing the availability of C_1_-compounds to improve methyl-H_4_F production increases the formation of methylated products in *E. coli*. [28] We surmised that the C_1_-compound formate could be an ideal supplier of C_1_-building blocks to fuel the C_1_-H_4_F metabolism of biotechnological hosts and to drive whole-cell methylation forward.

In previous publications, strategies to assimilate formate into the metabolism of *E. coli* have been established [13, 34]. Introduction of the formate-H_4_F ligase from *Methylorubrum extorquens* (*Mex*FTFL) into *E. coli* allows synthesis of formyl-H_4_F from formate and H_4_F in an adenosine-5´-triphosphate (ATP)-dependent reaction (Fig. 2a) [13, 35, 36]. Further, introduction of formyl-H_4_F cyclohydrolase (*Mex*FCH) and methylene-H_4_F dehydrogenase A (*Mex*MTDA), both from *M. extorquens*, cyclise and reduce formyl-H_4_F to methylene-H_4_F, the central metabolite of the C_1_-H_4_F metabolism, which can be used to further introduce formate’s carbon into central metabolites [35–37]. This formate assimilation strategy composed of *Mex*FTFL, *Mex*FCH and *Mex*MTDA (together referred to as FCM) proved powerful and was used for full assembly of all carbon scaffolds from formate and CO_2_ in synthetic formatotrophic *E.* *coli* (Fig. 2a) [13, 34, 38].

In order to harness this established formate assimilation strategy for SAM-dependent methylation, the generated methylene-H_4_F has to be reduced once more by the NADH-dependent methylene-H_4_F reductase (*Ec*METF, Fig. 2) to methyl-H_4_F [39]. *Ec*METE or *Ec*METH transfer the methyl group onto homocysteine to produce the proteinogenic amino acid methionine, the precursor of SAM [29]. Previously reported introduction of FCM in engineered *E. coli* strains allowed all methyl groups of methionine to derive from formate when ^13^C-labelled formate was fed, emphasising the potential to use formate for the synthesis of methyl groups [40]. Growth of the synthetic formatotrophs solely on formate is approximately 10-times slower than on established feedstocks [13, 38], making a strategy with growth from established feedstocks and supply of formate to push methylation of selected building blocks an interesting option.

To activate the methyl group, native methionine adenosyltransferases (*Ec*MAT, Fig. 2b) convert ATP and methionine to SAM as a first step of the SAM regeneration cycle [41]. The cofactor is subsequently utilised by MTs for methylation of their substrate, thereby, introducing the C_1_-compound into the product. Byproduct of the methylation reaction is *S*-adenosylhomocysteine (SAH), a known inhibitor of MTs [14]. In native *E. coli* metabolism, SAH is degraded in an irreversible reaction to adenine and *S*-ribosylhomocysteine by methyl thioadenosine/SAH nucleosidase (*Ec*MTAN) [42]. *S*-ribosylhomocysteine is cleaved by *S*-ribosylhomocysteine lyase (*Ec*LUXS) to homocysteine and the quorum sensing precursor 4,5-dihydroxypentane-2,3-dione (DHPD) [43]. Another option to degrade SAH are SAH hydrolases (SAHH, Fig. 2b) that cleave SAH to adenosine and homocysteine [44]. Transfer of a methyl group from methyl-H_4_F regenerates methionine from homocysteine allowing repetition of methylation with another C_1_-building block.

In order to monitor transformation of formate into the methyl groups of MT-derived products, a model MT reaction is needed, such as the anthranilate *N*-MT from *Ruta graveolens* (*Rg*ANMT, Fig. 2b). *Rg*ANMT transfers a methyl group in a SAM-dependent reaction onto the amino group of the unnatural substrate 2,5-aminonitrophenol (2,5-ANP) producing *N*-methyl-2,5-ANP (M-2,5-ANP, Fig. 2b) [26, 45]. The unnatural substrate and product undergo minimal degradation in *E. coli* and can freely permeate in and out of the bacterial cell, making this system a well-suited model for investigations on improving whole-cell methylation. Conversion rates of the model MT-substrate system were shown to be strongly dependent on the intracellular availability of methionine, resulting in a great potential to use it to explore the influence of formate on whole-cell methylation [26].

Recent research towards the application of formate mainly focussed on the incorporation of formate-derived carbon into central metabolites, either to allow growth or the production of small organic compounds [13, 46]. Reports on improving whole-cell methylation, on the contrary, focus on improving efficiency of the SAM regeneration cycle and intracellular availability of methionine, mostly omitting the supply of C_1_-units from the C_1_-H_4_F metabolism [23, 25, 26, 47]. In this study we combine these two separated fields of research to investigate formate as a supplementary source of C_1_-units to increase regeneration of methionine and, therefore, whole-cell methylation with *E. coli*. We first investigated the sole whole-cell biotransformation of formate into transferred methyl groups using the described formate assimilation machinery FCM in combination with different MTs. We then continued using engineered *E. coli* strains (Fig. 2c) to allow higher formation of a methylated product by the supplementation of formate.

## Methods

### Chemicals

All chemicals were purchased in the highest available purity from certified vendors: ^13^C-formate (> 99% ^13^C-enriched) was obtained as sodium formate from Sigma Aldrich, unlabelled sodium formate was obtained from Carl Roth chemicals; both were according to our knowledge not synthesised by electrochemical reduction. 2,5-Aminonitrophenol (2,5-ANP), 2,4-methoxy nitroaniline (2,4-MNA), 2,7-dihydroxynaphtalene (DHN), perchloric acid (70%), and formic acid (LC–MS grade) were purchased from Sigma Aldrich; NaCl, NH_4_Cl, MgSO_4_, KH_2_PO_4_, K_2_HPO_4_, CaCl_2_, agarose, LB-agar, LB-medium, d-( +)-glucose, d-( +)-xylose, glycerol, *iso*-propyl-β-thiogalactopyranosid (IPTG), kanamycin, chloramphenicol, and glycine were purchased from Carl Roth; 2 × Phusion DNA polymerase master mix, acetonitrile (HPLC grade or LC–MS grade) were purchased from Thermo Fisher Scientific. Ethidium bromide was purchased from Merck KGaA, In-Fusion 5 × master mix was purchased from Takara Bio Inc.; high fidelity restriction enzymes, CutSmart buffer, T4-DNA Ligase, 10 × T4-DNA ligase buffer, and 1 kb Plus DNA ladder were purchased from New England Biolabs; spectinomycin was purchased from VWR International.

### Strains and plasmids

See Table 1.

**Table 1:** Strains and plasmids used in this study (Gene sequences are given at the end of the SI)

Name	Genotype	Source
*E. coli* strains		
Stellar	*F–, endA1, supE44, thi-1, recA1, relA1, gyrA96, phoA, Φ80d lacZΔ M15, Δ (lacZYA—argF) U169, Δ (mrr—hsdRMS—mcrBC), ΔmcrA, λ–*	Takara Bio Inc.,Cat No. 636766
BL21(Gold)DE3	*F–ompT hsdS(rB– mB–) gal dcm λ(DE3)*	Novagen, Cat. No. 69450
ST18	*S17 λpir (pro thi hsdR*^+^ *Tp*^*r*^* Sm*^*r*^*; chromosome::RP4-2 Tc::Mu-Kan::Tn7/λpir) ∆hemA*	DSM 22074 [48,49]
SIJ488	*E. coli K-12 MG1655Tn7::para-exo-beta-gam; prha-FLP; xylSpm-IsceI*	[50]
BL21-AI (only used transfer *araB::T7RNAP-tetA)*	*F- ompT hsdSB(rB—mB -) gal dcm ΔaraB::T7RNAP-tetA*	Invitrogen (Cat. No. 11540146)
BL21(Gold) DE3*Δmetj*	*F–ompT hsdS(rB– mB–) gal dcm λ(DE3), ΔmetJ*	[22]
C_1_-Aux (C1A∆3)	SIJ488 *ΔgcvTHP*, *ΔglyA*, *ΔfrmRAB*	[51]
C_1_-opt	C1-Aux *ΔpflAB*, *ΔaraB::T7RNAP*, *fcm*	This study
C_1_S	SIJ488 *ΔgcvTHP, ΔserA, ΔfrmRAB, ΔecaraB::T7RNAP, fcm*	This study
C_1_-opt-*rganmt*	C_1_-opt harbouring pET22b*::rganmt*	This study
C_1_S-*rganmt*	C_1_S harbouring pET28a*::rganmt*	This study
C_1_S-*rganmt* + empty	C_1_S harbouring pET28a*::rganmt*, pCDF-Duet-1	This study
C_1_S-*rganmt* + *ecmat*	C_1_S harbouring pET28a*::rganmt*, pCDF*::ecmat*	This study
C_1_S-*rganmt* + *ecmtan* + *ecluxs*	C_1_S harbouring pET28a*::rganmt*, pCDF*::ecmtan_ecluxS*	This study
C_1_S-*rganmt* + *ecmat* + *ecmtan* + *ecluxS*	C_1_S harbouring pET28a*::rganmt*, pCDF*::ecmat_T7_ecmtan_ecluxS*	This study
C_1_S-*rganmt* + *ecmetF*	C_1_S harbouring pET28a*::rganmt*, pCDF*::ecmetF*	This study
BL21-*fcm-rganmt*	BL21(GOLD)DE3 harbouring pFCM and pET28a*::rganmt*	This study
BL21-*fcm-rganmt*-*ecmetF*	BL21(GOLD)DE3 harbouring pFCM, pET28a*::rganmt_ecmetF*,	This study
BL21-*fcm-rganmt*-*mmsahh*	BL21(GOLD)DE3 harbouring pFCM, pET28a*::rganmt_mmsahh*	This study
Plasmids		
pET28a( +)	*Kan*^*R*^*, lacI, T7lacl, pBR322, RBS*_*T7*_	Novagen, Cat. No. 69864
pET22b( +)	*Amp*^*R*^*, lacI, T7lacl, pBR322, RBS*_*T7*_	Novagen, Cat. No. 69744
pCDF-DUET-1	*Str*^*R*^*, lacI, T7lac, CloDF13, RBS*_*T7*_	Novagen, Cat. No. 71340
pKD3	*Amp*^*R*^* Cam*^*R*^*, carries chloramphenicol resistance gene flanked by FRT (flippase recognition target) sites*	[52]
pKD4	*Amp*^*R*^* Kan*^*R*^*, carries kanamycin resistance gene flanked by FRT (flippase recognition target) sites*	[52]
pDM4	*Kan*^*R*^*, Cam*^*R*^, *sacB*, *traJI*, *oriR6K*	[53,54]
pET28a*::rganmt*	pET28a( +) vector containing *rganmt*	[26]
pET22b*::rganmt*	pET22b( +) vector containing *rganmt*	This study
pET28a*::rganmt_ecmetF*	pET28a( +) vector containing *rganmt, rbs, ecmetF*	This study
pET28a*::rganmt_mmsahh*	pET28a( +) vector containing *rganmt, rbs, mmsahh*	This study
pCDF*::ecmat*	pCDF-DUET-1 vector containing *ecmat* in MCS-1	[26]
pCDF*::ecmtan_ecluxS*	pCDF-DUET-1 vector containing *ecmtan, rbs, ecluxS* in MCS-1	[26]
pCDF*::ecmat_T*_*7_*_*ecmtan_ecluxS*	pCDF-DUET-1 vector containing *ecmtan* in MCS-1 and *ecmtan, rbs, ecluxS* in MCS-2	[26]
pCDF::*ecmetF*	pCDF-DUET-1 vector containing *ecmetf* in MCS-1	This study
pFCM	pSC101 ori, *Strep*^*R*^, *P*_*pgi-mut*_, *mexftfl*, *mexfch*, *mexmtda*	[40]
pDM4:SS9-C_1_M	pDM4 vector containing 600 bp up- and downstream homology to safe spot 9^[[55]]^ for the genome integration of *fcm* (*P*_*STRONG*_*-RBS*_*C*_*-ftfl-RBS*_*C*_*-fch-RBS*_*C*_*-mtdA*)	[13]

### *E. coli* strain engineering

All strains and plasmids used in this study are listed in Table 1. The strain C_1_-opt was constructed from the published C_1_-Aux (C_1_A∆3) strain based on *E. coli* strain SIJ488 [50], a derivative of MG1655, engineered to carry inducible genes for λ-Red recombineering and flippase in its chromosome and C_1_S was directly constructed from *E. coli* strain SIJ488 [50]. Genome engineering was performed by λ-red recombineering [52] and P1 phage transduction [50,52]. In brief, the genes indicated in Table 1 for C_1_S and C_1_-opt were deleted using PCR-amplified linear DNA fragments carrying kanamycin or chloramphenicol resistance markers flanked by FRT-sites based on the pKD4 or pKD3 plasmids, respectively [52]. Resistance markers were amplified with primers carrying 50 bp overhangs serving as homologous regions flanking the target gene [56]. For removal of resistance markers from the chromosome, flippase was induced by addition of l-rhamnose as described previously [50]. Gene deletions and removal of resistance cassettes were verified by PCR.

For chromosomal insertion of the genes encoding the enzymes for formate assimilation (*Mex*FTFL, *Mex*FCH and *Mex*MTDA, together referred to as FCM) [13,34], we used a previously described genome insertion protocol [53] and vector [13] containing the desired genes (“pDM4:SS9-C_1_M”, Table 1). In brief, a non-replicative plasmid (pDM4, R6K ori) was introduced into the respective recipient strain via conjugation from an *E. coli* ST18 donor strain, as described previously [48,49,53]. Kanamycin resistance was used to select for chromosomal insertion of the synthetic FCM operon (including the kanamycin resistance gene) via native homologous recombination based on 600 bp homology regions, with subsequent levansucrase (*sacB*) counter-selection.

For chromosomal insertion of the arabinose-inducible T7-RNA polymerase to create C_1_S and C_1_-opt, we transferred the corresponding genomic locus from BL21-AI (Invitrogen) using P1 phage transduction. The transferred locus consists of an insertion replacing the *araB* gene, introducing genes encoding a tetracycline efflux pump (a selectable tetracycline resistance marker) and T7-RNA polymerase [57]. The latter is controlled by the endogenous P_araBAD_ promoter, thus, achieving arabinose-inducible expression of T7-RNA polymerase. Successful transductants were selected via tetracycline resistance and the insertion site was verified by PCR.

### Plasmid construction

C_1_-opt already contains a kanamycin resistance, which is why the anthranilate *N*-MT from *Ruta graveolens* (*rganmt*) was cloned with the restriction enzymes *Nde*I and *Hind*III into an ampicillin resistance-containing pET22b( +) vector: Separately, 1 µg of the vectors pET28a*::rganmt* and pET22b( +) were digested with *Nde*I and *Hind*III at 37 °C for 2 h followed by separation by agarose gel electrophoresis (1% agarose, 100 V, 50 min) and clean-up of the excised bands of the cut *rganmt* insert and the cut pET22b( +) vector using the Promega Wizard^™^ Plus DNA Purification System. 50 ng of the linear vector were combined in a 1:3 ratio with the respective gene to be inserted and 2 µl of 5 × In-Fusion buffer in a total volume of 10 µl nuclease free water. The mixture was incubated at 50 °C for 20 min followed by standard chemical transformation into *E. coli* Stellar cells, as recommended for the Takara Bio In-Fusion Cloning. The identity of each vector was confirmed by sequencing.

For co-overexpression on pET28a vector with BL21, the *ecmetF* gene and the SAH hydrolase gene from *Mus musculus* (*mmsahh*) were cloned behind *rganmt* by digesting pET28a*::rganmt* with *Xho*I and cloning of the respective insert into the linearised vector using the seamless In-Fusion Cloning technique (Table 2)*.* When co-overexpression was conducted with pET28a and pCDF-DUET-1 vector in C_1_S, *ecmetF* was cloned into the first MCS of pCDF-DUET-1 (Table 2). For this purpose, the respective inserts were amplified from a pET28a vector and the pCDF-DUET-1 vector was linearised via polymerase chain reaction (PCR, Table 2):

**Table 2:** Utilised oligonucleotides

Utilisation	Sequence
Linearisation of pCDF DUET-1 MCS-1 slot *fwrd*	TCTACTAGCGCAGCTTAA
Linearisation of pCDF DUET-1 MCS-1 slot *rev*	TATGTATATCTCCTTCTTATACTT
Cloning of a gene from pET28a into MCS-1 of pCDF-DUET-1 *fwrd*	CTTTCTGTTCGACTTGCCGGATCTCAGTGGTGG
Cloning of a gene from pET28a into MCS-1 of pCDF-DUET-1 *rev*	AAGGAGATATACCATAAGAAGGAGATATACCATGGGCAGC
Cloning of *ecmetF* or *mmsahh* behind *rganmt* in pET28a after digestion with *Xho*I* fwrd*	GGTGGTGGTGCTCGATTTGTTAGCAGCCGGATCTCAG
Cloning of *ecmetF* or *mmsahh* behind *rganmt* in pET28a after digestion with *Xho*I* rev*	TGCGGCCGCACTCGATTAAGAAGGAGATATACCATGGGC
Constitutive promoter *P*_*pgi-mut*_ from pFCM [40]	GTGGAATTATAGCATTTTAGCCTTTAATTGTCAATAGGTCTCGAGGTGAAGACGAAAGGGCCTCG
Constitutive promoter *P*_*STRONG*_ from pDM4:SS9-C_1_M [13,53,58]	AATACTTGACATATCACTGTGATTCACATATAATATGCG
*RBS*_*C*_ [53]	AAGTTAAGAGGCAAGA

Primers with 15 bp overhangs complementary to the vector were ordered at Eurofins as lyophilised powder. The PCR was set up with 3 µl fwrd primer (c = 10 pM), 3 µl rev primer (c = 10 pM, Table 2), 1 µl of template (pET28a vector with the gene of interest or yeast gDNA), 18 µl nuclease free water, and 25 µl 2 × Phusion master mix (Thermo Fisher). The PCR was conducted with 5 min initial denaturation at 98 °C, followed by 29 cycles of 20 s denaturation at 98 °C, 20 s annealing at 55 °C and 35 s elongation at 72 °C. Afterwards, 5 min terminal elongation at 72 °C followed. The size of the gene was confirmed by agarose gel electrophoresis (1% agarose, 100 V, 50 min), followed by extraction of the band and clean up with the Promega Wizard^™^ Plus DNA Purification System. For insertion of the gene, the vectors were linearised with the respective primer pairs or with *Xho*I (Table 2). 50 ng of the linear vector were combined in a 1:3 ratio with the respective gene to be inserted and 2 µl of 5 × In-Fusion buffer in a total volume of 10 µl nuclease free water. The mixture was incubated at 50 °C for 20 min followed by standard chemical transformation into *E. coli* Stellar cells, as described for the Takara Bio In-Fusion Cloning. Identity of each vector was confirmed by sequencing.

### Assay procedure to perform whole-cell methylation

For whole-cell methylation experiments, the *E. coli* strains were chemically (co-)transformed with the respective plasmids and then grown overnight at 37 °C on agar plates supplemented with the required antibiotics. A single colony was picked and used to inoculate 5 ml LB-medium (+ antibiotic) followed by overnight incubation at 37 °C, 170 rpm. The preculture was used to inoculate 25 ml LB-medium (+ antibiotic) in a 50 ml falcon tube in a ratio of 1:100. The culture was grown to an OD_600_ of 0.5 – 0.7, overexpression was induced with 1 mM IPTG and took place at 20 °C, 140 rpm for 20 h (the lower temperature and shaking velocity was chosen to lower the stress level during protein production).

After overexpression, the cultures were chilled on ice for 10 min followed by centrifugation for 15 min, 4 °C, 2,500 g. The pellet was washed with 5 ml of ice cold M9-medium (48 mM Na_2_HPO_4_, 22 mM KH_2_PO_4_, 8.6 mM NaCl, 1.8 mM NH_4_Cl, 2 mM MgSO_4_, 0.1 mM CaCl_2_, and 22 mM glucose as carbon source. NOTE: for experiments conducted for Figure SI 5 & SI 6 20 mM glycerol or 13 mM xylose were used as carbon source instead) followed by centrifugation (15 min, 4 °C, 2,500 g). The pellet was then resuspended to an OD_600_ of approximately 4.5 in ice cold M9-medium containing 1 mm IPTG and required antibiotics. Assays were then pipetted on ice in 1.5 ml reaction tubes in a total volume of 1 ml followed by incubation at 37 °C and 170 rpm for 24 h. The whole-cell catalysts were used in a final OD_600_ of 3.0 with 0.75 mm of the substrate to be methylated (2,5-ANP or DHN), 2.5–75 mm ^13^C-formate and optionally 2 mm glycine. To terminate the assay after 24 h, 100 µl of the assay mixture were taken and mixed with 35 µl of 10% perchloric acid followed by centrifugation at 4 °C and 18,000 g for 30 min. The supernatant was taken and stored at −20 °C until analysis. For determination of conversion rates, HPLC–UV (λ = 280 nm) analysis was used and the share of ^13^C-labelled methylated product was elucidated with LC–MS/MS.

### Statistics

A t-test was performed to compare the mean product formation within 24 h across different conditions. A two-sided independent samples t-test assuming equal variances was applied. Statistical significance was defined as p < 0.05. All experiments were conducted in at least three biological replicates.

### HPLC–UV analysis

HPLC–UV analysis (λ = 280 nm) was used to detect MT substrates and products generated during whole-cell methylation experiments and to calculate product formation. For analysis, 10 µl of the sample were injected into an Agilent 1260 infinity II System equipped with a quaternary pump and the samples were separated on an ISAspher 100-3-C18 column (150 × 4.0 mm) with an elution gradient method using 0.1% formic acid in MilliQ water as mobile phase A and acetonitrile as mobile phase B at a flow rate of 1.0 ml *min^−1^ (s. Table SI 2). The method was initiated at 3% mobile phase B and held for 4 min, followed by an increase to 70% mobile phase B within the next 3 min. Within the following 0.1 min, mobile phase B was increased to 100% and kept at this level for another 2.9 min, followed by a decrease to 3% mobile phase B within 1 min and equilibration for another 3 min. Conversion was calculated by Eq. 1.

Equation 1. Calculation of conversion to methylated products from HPLC-peak area (area under curve, AUC).

1 $${\text{Conversion}} \,\left[ {\text{\%}} \right]{ = }\, \frac{{\text{AUC\,(product)}}}{{{\text{AUC}} \,\left( {{\text{product}}} \right){\text{ + AUC (substrate)}}}}\,{*}\,{100}$$

### LC–MS/MS analysis

LC–MS/MS was used for analysis of the share of ^13^C-labelled products. For analysis, 1 µL of the 1:10 diluted sample was injected using a SCIEX 5500 + triplequad LC–MS/MS and the samples were separated on an ISAspher 120-3-C18 Aq column (50 × 2.1 mm) with the same mobile phases and elution gradient method as for HPLC–UV analysis but with a flow rate of 0.2 ml *min-1 (s. Table SI 3). MS conditions and mass shifts were used as described in Table SI 4. The share of ^13^C-labelled product was calculated by Eq. 2.

Equation 2. Calculation of share of ^13^C-labelled product from LC–MS/MS peak area (area under curve, AUC).

2 $${\text{Share }}\left[ {\text{\%}} \right] = { }\frac{{{\text{AUC }}(^{13} {\text{C}} - {\text{prod}})}}{{{\text{AUC }}(^{12} {\text{C}} - {\text{prod}}) + {\text{AUC }}(^{13} {\text{C}} - {\text{prod}})}}{*}100$$

## Results

### *N*-Methylation with formate-derived methyl groups using *E. coli* BL21-*fcm-rganmt*

We started by chemical transformation of *E. coli* BL21 (Gold)DE3 (abbreviated as BL21 in the following) with a plasmid carrying the *fcm* genes under the control of a constitutive promoter (pFCM, s. Table 1) and a pET28a vector carrying the *rganmt* gene, resulting in BL21*-fcm-rganmt* (Table SI 1). To analyse conversion rates, a biotransformation approach was chosen, where the MT is first overproduced together with FCM in rich medium (lysogeny broth, LB) during the overexpression phase, followed by harvest, and resuspension in glucose containing minimal medium (M9) to normalise the cell densities (final OD_600_ = 3.0) and allow energy regeneration. Subsequently, the biotransformation phase is initiated by adding the methyl acceptor substrate (2,5-ANP) for the methylation reaction together with formate [59,60].

**Fig. 3:**
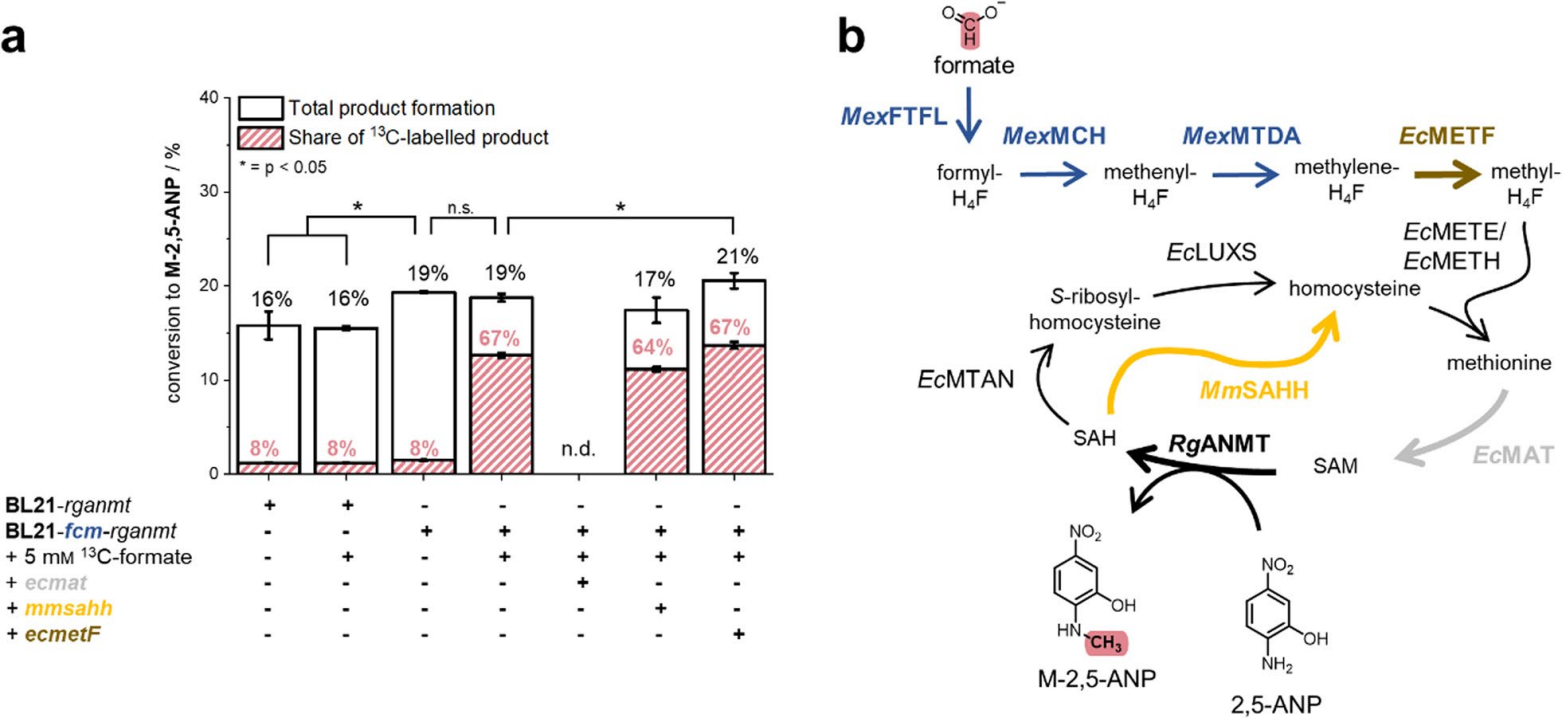
Whole-cell methylation with BL21-*rganmt* supplying ^13^C-formate. **a** Given is the conversion of 2,5-ANP to M-2,5-ANP (black) and the share of ^13^C-labelled product ± SD (red) under different conditions and co-overexpression of genes involved in formate to methyl group transformation or SAM regeneration. **b** Scheme of the metabolic pathway from formate to the methylated product with overproduced enzymes highlighted. Experiments were performed in biological triplicates. n.d. = not detected. Experiments were conducted in M9-medium (22 mm glucose) with a final OD_600_ of 3.0, 0.75 mM 2,5-ANP and 0–5 mM ^13^C-formate at 37 °C, 170 rpm for 24 h. NOTE: About 8% of M-2,5-ANP is naturally ^13^C-labelled, therefore shares given are indicators not absolutes for formate-derived methyl groups. *2,5-ANP* - 2,5-aminonitrophenol, *H*_*4*_*F* - tetrahydrofolate, *M-2,5-ANP - N*-methyl-2,5-aminonitrophenol, *SAM - S*-adenosylmethionine, *SAH - S*-adenosylhomocysteine, *EcLUXS - S*-ribosylhomocysteine lyase from *E. coli*, *EcMAT* - methionine adenosyltransferase from *E. coli*, *EcMETE/EcMETH* - methionine synthase from *E. coli*, *EcMETF* - methylene-H_4_F reductase from *E. coli*, *EcMTAN - S*-methyl-5´-thioadenosine/SAH nucleosidase from *E. coli*, *MexFTFL -* formate-H_4_F ligase from *M. extorquens*, *MexMCH* - formyl-H_4_F cyclohydrolase from *M. extorquens*, *MexMTDA* - methylene-H_4_F dehydrogenase A from *M. extorquens*, *MmSAHH -* SAH hydrolase from *M. musculus*, *RgANMT -* anthranilate *N*-MT from *R. graveolens.*

Comparison of BL21*-fcm-rganmt* to a strain without formate assimilation machinery (BL21-*rganmt*) showed that introducing pFCM already improves product formation without addition of formate (Fig. 3). A similar increase in product formation was observed when biotransformation experiments were conducted with BL21-*rganmt* in combination with an empty pCDF-DUET-1 vector carrying a spectinomycin resistance in a previous study [26]. Accordingly, the increase in product formation detected here is suggested to be caused through introduction of the spectinomycin resistance encoded on pFCM, an adenylyltransferase [26]. Hereby enhanced ATP regeneration increases ATP availability for SAM synthesis and, hence, improves product formation [26]. Supplementation with 5 mM ^13^C-formate did not yield higher conversion for BL21-*rganmt* or BL21-*fcm*-*rganmt* (Fig. 3). Analysis with LC–MS/MS, however, revealed that 67% of the produced M-2,5-ANP were ^13^C-labelled in the experiment with BL21-*fcm*-*rganmt* (Fig. 3, Figure SI 1). This incorporation of formate-derived methyl groups did not take place in BL21-*rganmt* highlighting the necessity of pFCM for the assimilation of formate.

Despite introduction of formate-derived methyl groups and the, therefore, increased availability of C_1_-building blocks in BL21*-fcm-rganmt* fed with ^13^C-formate, conversion rates did not improve as expected from literature reports [28]. As shown in our previous study, supplementation of BL21-*rganmt* with 5 mM methionine strongly increased product formation; therefore, neither MT acceptor substrate permeation into the bacterial cell, nor activity of *Rg*ANMT seemed to be the major limiting factors. We rather suspected a kinetic bottleneck either in the SAM regeneration cycle or in the reduction of methylene-H_4_F to methyl-H_4_F through *Ec*METF. We co-overexpressed *ecmat* and the SAH hydrolase gene from *Mus musculus* (*mmsahh*), which should improve SAH degradation through cleavage to adenosine and homocysteine; as well as the *ecmetF* gene together with *rganmt* encoded on a pET28a vector. Curiously, BL21-*fcm*-*rganmt*-*ecmat* did not produce any methylated product. An increased metabolic burden through overproduction of two enzymes could be one reason for the loss of methylation activity of this strain. Co-overexpression of *mmsahh* in BL21-*fcm*-*rganmt*-*mmsahh* lowered conversion rates slightly in combination with a decreased share of labelled methylated product. A small increase in conversion with BL21-*fcm*-*rganmt-ecmetF* (21% conversion) supplemented with 5 mM ^13^C-formate compared to BL21-*fcm*-*rganmt* (19% conversion) was observed (Fig. 3). Reduction of methylene-H_4_F to methyl-H_4_F by *Ec*METF, hence, seems to be a minor bottleneck for whole-cell biotransformation of formate to methyl groups. Nevertheless, the increase in methylated product was rather small, which is why we omitted co-overexpression in the following experiments. These first results demonstrated straight-forward conversion of formate into transferable methyl groups by whole-cell biotransformation using *E. coli*.

### Chemoselective *C*- and *O*-methylation with formate-derived methyl groups

We further analysed, whether other MTs could also be used to methylate with formate-derived SAM. The caffeic acid *O*-MT from *Prunus persica* (*Pp*CaOMT) also accepts 2,5-ANP, however, catalyses an *O*-methylation to form 2,4-methoxy nitroaniline (2,4-MNA, Figure SI 2). While classic chemical methods struggle to selectively *N*- or *O*-methylate 2,5-ANP, the here described MTs were demonstrated to be highly selective [45]. As a third model reaction, the *C*-MT from *Streptomyces rishiriensis* (CouO) was chosen for the transfer of a methyl group onto the unnatural substrate 2,7-dihydroxynaphtalene (DHN), producing 1-methyl-DHN (M-DHN, Figure SI 2). The selectively methylated, functionalised MT-products are interesting as building blocks for chemical synthesis of small molecules such as active pharmaceutical ingredients. After the biotransformation experiment with supply of 5 mM ^13^C-formate, shares of ^13^C-labelled 2,4-MNA and M-DHN were 51% and 81%, respectively; again, without a substantial change in conversion rates (Figure SI 2). In a previous study, co-overproduction of *Ec*MAT and CouO was found to strongly increase conversion rates; this was also the case here: Co-overproduction of *Ec*MAT together with FCM and CouO increased product formation about 3.0-fold with and without the addition of formate. When 5 mM ^13^C-formate were supplied, a share of 68% of M-DHN was ^13^C-labelled (Figure SI 2). These results show the flexible applicability of whole-cell methylation with formate-derived methyl groups, using BL21-*fcm* in combination with different MTs.

### Selecting engineered *E. coli* strains for improving methylation through the supply of formate

Introduction of formate-derived carbon into methyl groups worked successfully; however, conversion rates did not improve by the supply of formate. Initially, we suspected regulation through the transcription repressor *Ec*METJ to hamper reduction of formate to methyl groups by decreasing the expression of *ecmetF*, *ecmetE*, *ecmetH*, *ecmat* and genes involved in the synthesis of homocysteine [61,62]. Experiments with BL21*ΔmetJ*-*fcm* in combination with the MTs did not result in an increase of conversion rates when formate was supplemented (Figure SI 3). Furthermore, growth of the cultures and overall conversion decreased in comparison to BL21-*fcm*, suggesting that BL21*ΔmetJ*-*fcm* was not the optimal host to continue the investigations of transforming formate into methyl groups. Instead, we engineered strains based on *E. coli* K-12 MG1655. The strains’ design was performed in accordance with the results of a previous study where *Yishai et al.* [40] (i) introduced the *fcm* genes on a plasmid under control of a constitutive promoter, and (ii) deleted genes involved in the native generation of C_1_-H_4_F compounds, leading to excellent results in converting formate into the methyl group of methionine [40]. *Yishai et al.* achieved a C_1_-auxotrophy in *E. coli* by deletion of the glycine cleavage system (*Ec*GCS, encoded by *ecgcvTHP*) and serine hydroxymethyltransferase (*ecglyA*), which represent the main supply pathways for C_1_-H_4_F metabolites, forcing assimilation of formate for the generation of C_1_-H_4_F compounds [40]. In this study, *ecglyA* and *ecgcvTHP* were deleted to construct the C_1_-auxotrophic strain C_1_-opt. Additionally, the serine-C_1_ auxotrophic strain C_1_S was constructed, in which *ecgcvTHP* was deleted, *ecglyA* is still intact but the *de novo* biosynthesis of serine is not possible due to deletion of the 3-phospho-glycerate dehydrogenase (*ecserA*) [40]. For both strains, formate assimilation is enabled by genomic introduction of the *fcm* genes under control of a constitutive promoter (*P*_*STRONG*_, Table 1), a genomic introduced arabinose-inducible T7 RNA-polymerase system allows overproduction of MTs from the pET28a vector (Fig. 2, Table 1) [57].

**Fig. 4:**
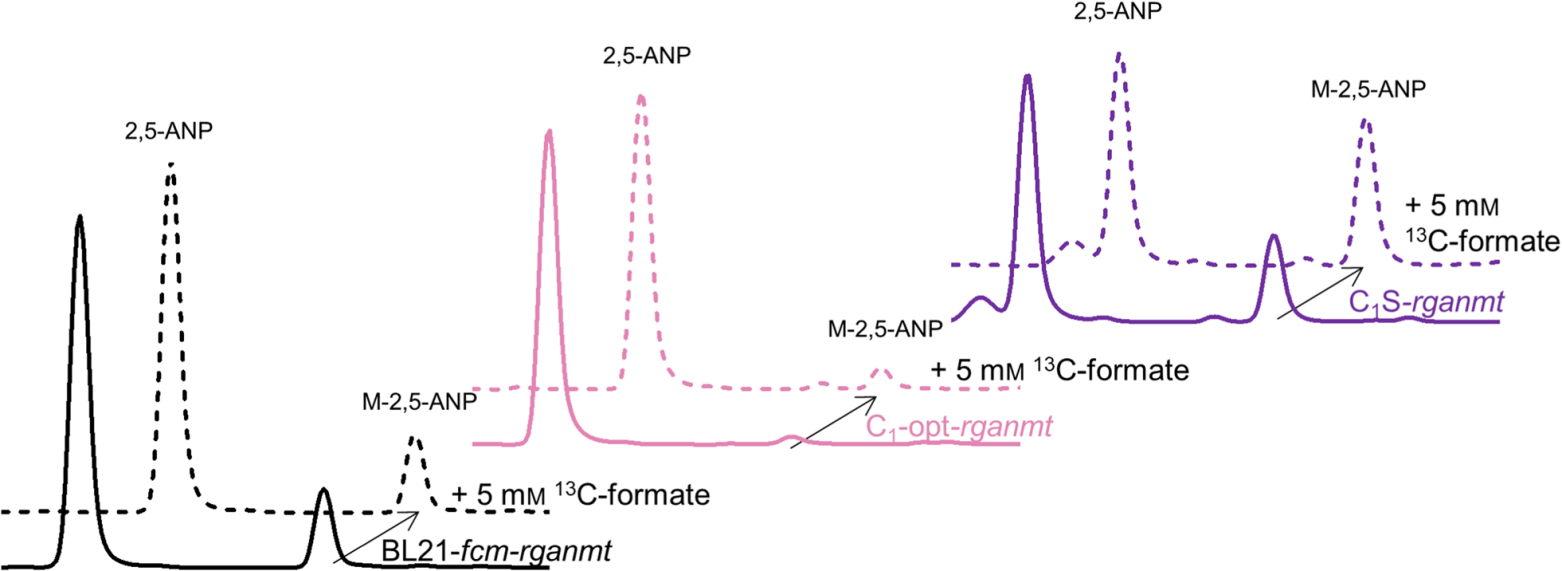
Whole-cell methylation by BL21-*fcm-rganmt*, C_1_-opt-*rganmt* and C_1_S-*rganmt*. HPLC–UV (λ = 280 nm) chromatogram of whole-cell methylation experiments using BL21-*fcm-rganmt*, C_1_-opt-*rganmt* and C_1_S-*rganmt* without (unbroken line) and with the addition of 5 mm ^13^C-formate (dotted line). Experiments were conducted in M9-medium (22 mM glucose) with a final OD_600_ of 3.0, 0.75 mM 2,5-ANP, and 5 mM ^13^C-formate at 37 °C, 170 rpm for 24 h. *2,5-ANP * - 2,5-aminonitrophenol, *M-2,5-ANP* - *N*-methyl-2,5-aminonitrophenol, *fcm* formate assimilation machinery genes from *M. extorquens*, *rganmt* - anthranilate *N*-MT gene from *R. graveolens*

To elucidate basic conversion rates, we focussed on *N*-methylation with *Rg*ANMT and compared BL21-*fcm-rganmt*, C_1_-opt-*rganmt* and C_1_S-*rganmt*. Following the overexpression phase in LB-media, all strains methylate 2,5-ANP to M-2,5-ANP without the addition of formate as analysed with HPLC–UV and LC–MS/MS (Fig. 4, Table SI 1). Product formation with C_1_S-*rganmt* was 1.5-fold higher than with BL21-*rganmt*, while with C_1_-opt-*rganmt* only about 2% of the substrate were converted to the methylated product (Fig. 4, Table SI 1). Growth of the cultures in LB-medium presumably allowed a certain part of metabolites (*e.g.* methionine, SAM, or serine for C_1_S) to be carried over or reside in the cells enabling whole-cell methylation without the addition of formate. Supplying C_1_-opt-*rganmt* with 5 mM ^13^C-formate led to a twofold increase of methylation with a share of 54% ^13^C-labelled product. As conversion rates were still only about 5%, this strain was not further investigated, as it was unable to compete with BL21-*fcm-rganmt* in terms of share of formate-derived methyl groups or total product formation. Product formation of C_1_S-*rganmt* increased 1.4-fold (42% conversion) when 5 mM ^13^C-formate were added, clearly outperforming BL21-*fcm-rganmt-ecmetF* (21% conversion)*.* To our surprise, in the experiment with C_1_S-*rganmt* only 15% of M-2,5-ANP were ^13^C-labelled, indicating only a fraction of transferred methyl groups to be derived from formate. Due to the clear increase in conversion rate, a much higher share was expected. Still, in comparison to BL21-*fcm-rganmt,* whole-cell methylation with C_1_S-*rganmt* benefitted from the addition of formate which is why we continued focussing on this C_1_-auxotrophic strain.

### Improving methylation with formate-derived methyl groups using C_1_S-***rganmt***

**Fig. 5:**
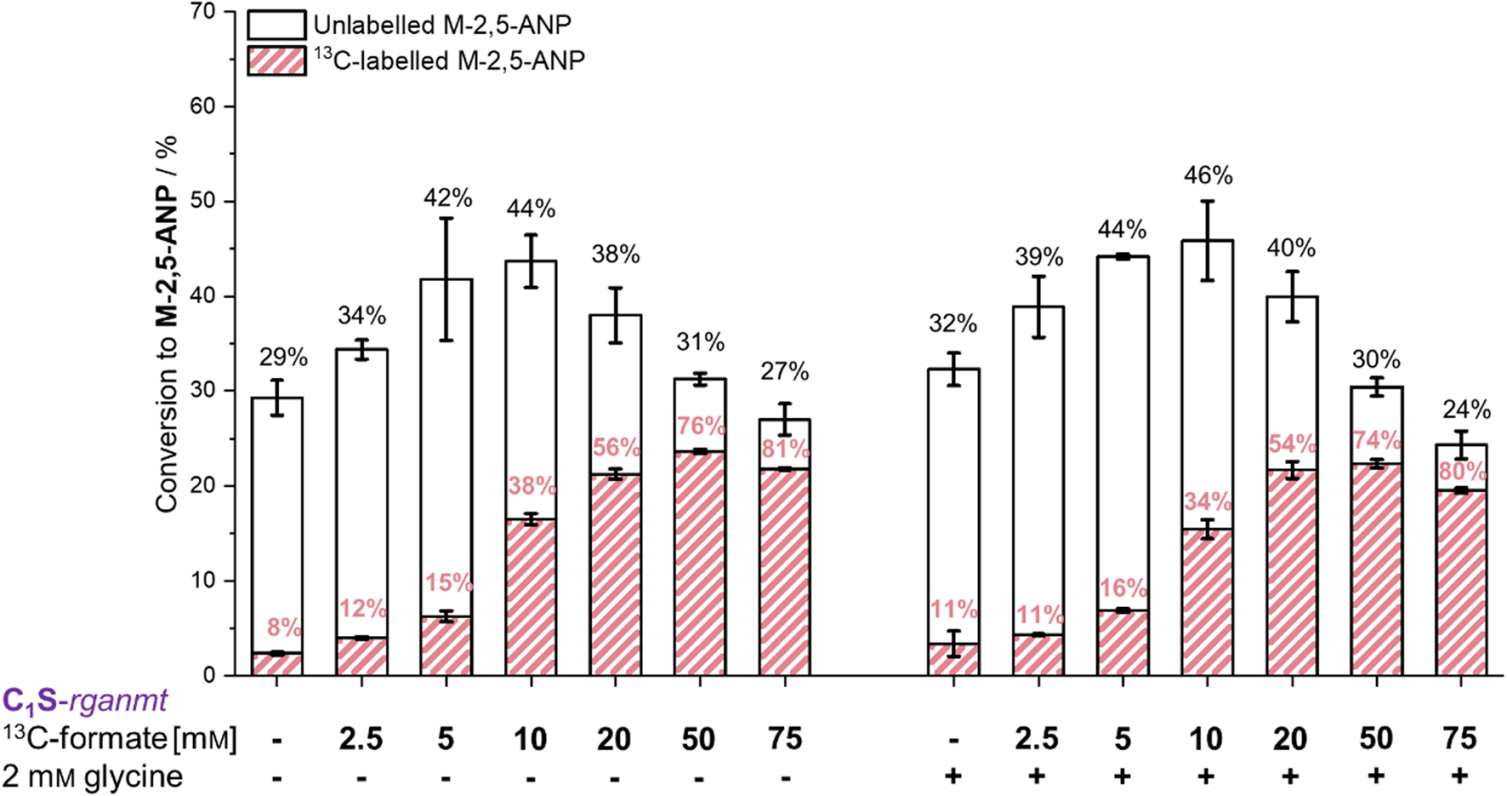
Increasing whole-cell methylation with formate in C_1_S-rganmt. Given is the conversion of 2,5-ANP to M-2,5-ANP by C_1_S-*rganmt* (black) and the corresponding share of ^13^C-labelled product (red) after supply with 2.5–75 mm ^13^C-formate with and without the addition of 2 mm glycine. Experiments were conducted in M9-medium (22 mm glucose) with a final OD_600_ of 3.0, 0.75 mm 2,5-ANP, 2.5–75 mm ^13^C-formate, and optionally 2 mm glycine at 37 °C, 170 rpm for 24 h. NOTE: About 8% of M-2,5-ANP is naturally ^13^C-labelled, therefore respective shares are indicators, not absolutes for formate-derived methyl groups. No significant difference was found between supply of the same formate concentration with and without the addition of glycine. *M-2,5-ANP* - *N*-methyl-2,5-aminonitrophenol, *rganmt* - anthranilate *N*-MT gene from *R. graveolens*

To evaluate the influence of formate concentration on product formation during the biotransformation phase, formate concentrations ranging from 2.5 mM to 75 mM were screened (Fig. 5, Figure SI 4). Substantial changes in conversion started from 2.5 mM and peaked at 10 mM ^13^C-formate supply with a 2.1-fold higher product formation compared to BL21*-fcm-rganmt*-*metF* supplemented with 5 mM ^13^C-formate (Table SI 1). Interestingly, shares of labelled product with 10 mM ^13^C-formate did not exceed 38%. Further increase of ^13^C-formate supply lowered product formation with C_1_S-*rganmt* whereas the shares of labelled product increased, indicating that the reported toxic effects of formate outweigh its benefit at such high concentrations [63,64]. When 50 mM ^13^C-formate were supplied, 31% conversion with 76% ^13^C-labelled methylated product were detected, giving the highest total amount of labelled product.

In the C_1_S strain, *Ec*GLYA is still present and could use methylene-H_4_F to synthesise serine from glycine when an excess of glycine is supplemented. On one hand, this reaction could withdraw C_1_-compounds from the C_1_-H_4_F metabolism, leading to decreased availability of C_1_-carbon units for whole-cell methylation. On the other hand, allowing serine synthesis could increase cysteine concentrations and through the transsulfuration pathway also methionine availability, supporting whole-cell methylation [65]. To test this, we repeated the screening with 2.5–75 mm ^13^C-formate in combination with supply of 2 mM glycine during the biotransformation phase in M9 media (Fig. 5). Co-feeding of ^13^C-formate and glycine increased conversion rates slightly (2–5% higher conversion, not statistically significant) for every condition up to 20 mm formate. With further increase of the C_1_-compound concentration, the small benefits of supplied glycine subsided. In most conditions, the share of ^13^C-labelled product was slightly lower when glycine was added, indicating that some of the ^13^C-labelled C_1_-compound was indeed withdrawn from the C_1_-H_4_F metabolism. While the differences observed were not statistically significant, the supply of 10 mm ^13^C-formate and 2 mM glycine still yielded the highest total conversion rate within this study (46% conversion with a share of 34% ^13^C-M-2,5-ANP). We assume that residing methionine from LB-media of the overexpression phase is responsible for the non-linear increase of labelled product with the addition of ^13^C-formate. Increasing the share of formate-derived methyl groups using moderate formate concentrations by exchanging media and carbon sources during overexpression did, however, not improve the total amount of formate-derived methyl groups (Figure SI 5 & Figure SI 6). We therefore focussed on improving product formation with C_1_S-*rganmt* and 50 mm ^13^C-formate to maintain a high incorporation of formate into the methylated product.

**Fig. 6:**
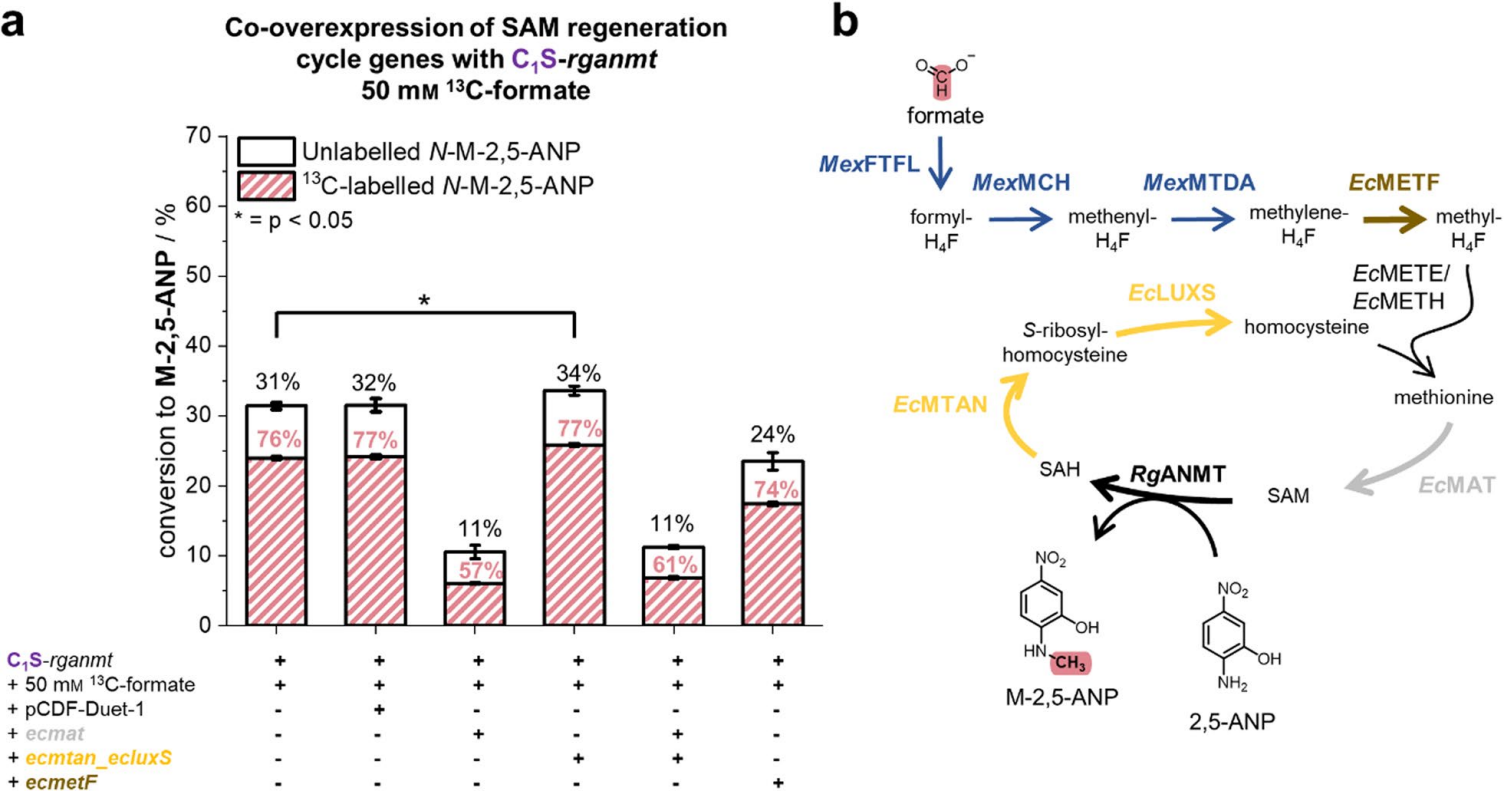
Co-overexpression of SAM regeneration cycle genes in C_1_S-*rganmt*. **a** Co-overexpression of *rganmt* and adjuvant genes encoded on a pCDF-DUET-1 vector in C_1_S. Given are the conversion rates (black) and the relative share of ^13^C-labelled product (red). **b** Scheme of the metabolic pathway from formate to methyl groups with co-overproduced proteins highlighted. Experiments were conducted in M9-medium, with 22 mm glucose, a final OD_600_ of 3.0, 0.75 mm 2,5-ANP and 50 mm ^13^C-formate at 37 °C, 170 rpm for 24 h. NOTE: About 8% of M-2,5-ANP is naturally ^13^C-labelled, therefore respective shares are indicators, not absolutes for formate-derived methyl groups. *p < 0.05, *2,5-ANP* - 2,5-aminonitrophenol, *H*_*4*_*F* - tetrahydrofolate, *M-2,5-ANP - N*-methyl-2,5-aminonitrophenol, *SAM - S*-adenosylmethionine, *SAH - S*-adenosylhomocysteine, *EcLUXS -* *S*-ribosylhomocysteine lyase from *E. coli*, *EcMAT* - methionine adenosyltransferase from *E. coli*, *EcMETE/EcMETH* - methionine synthase from *E. coli*, *EcMETF* - methylene-H_4_F reductase from *E. coli*, *EcMTAN - S*-methyl-5´-thioadenosine/SAH nucleosidase from *E. coli*, *MexFTFL -* formate-H_4_F ligase from *M. extorquens*, *MexMCH* - formyl-H_4_F cyclohydrolase from *M. extorquens*, *MexMTDA -* methylene-H_4_F dehydrogenase A from *M. extorquens*, *RgANMT -* anthranilate *N*-MT from *R. graveolens.*

To do so, the genes of the SAM regeneration cycle as well as *ecmetF* were co-overexpressed on a separate pCDF-DUET-1 vector in C_1_S-*rganmt* (Fig. 6, Figure SI 7). Increasing SAM synthesis by co-overexpression of *ecmat* lowered conversion, as was seen during the experiments with BL21-*rganmt*. Similar results were obtained for the co-overexpression of *ecmetF*. Co-overexpression of *ecmtan* and *ecluxS* led to a slight increase in product formation (~ 2% higher conversion), indicating that removal of SAH and regeneration to homocysteine could be a minor factor limiting whole-cell methylation in C_1_S-*rganmt*.

## Discussion

In this study we report that *E. coli* can be used to transform formate into methyl groups by introducing the formate assimilation machinery of *M. extorquens*. Introduction of formate-derived carbon into methylated products was highly efficient in BL21-*fcm*-*rganmt* supplied with 5 mM formate while conversion was not improved. Product formation with C_1_S-*rganmt*, in contrast, benefited from the addition of 5–10 mM formate, while the shares of formate-derived methyl groups peaked at 50 mM formate supply with slight decrease in conversion. Due to the energy-intensive production of renewable formate, low supply of the C_1_-compound is desirable, with the aim to (i) improve product formation and to (ii) incorporate formate-derived methyl groups into the products. The so far best performing strains C_1_S-*rganmt* or C_1_S-*rganmt-ecmtan*-*ecluxS* will be the starting point for future adjustments towards application.

We suspect carbon source-derived unlabelled C_1_-compounds to be the cause for the non-linear incorporation of formate-derived methyl groups with increased formate supply using C_1_S. Suppressing the formation of unlabelled C_1_-compounds should, hence, improve efficiency of whole-cell biotransformation of formate to methyl groups. One source of unlabelled formate could be the native pyruvate formate-lyase (*Ec*PFL). *Ec*PFL is an oxygen-sensitive enzyme and native expression of *ecpfl* occurs under anaerobic conditions providing a further electron sink for fermentation by cleavage of pyruvate to acetyl-CoA and formate [66]. Another possibility could be the production of intracellular formaldehyde from the added carbon source, which can be used by the unspecific threonine aldolase (*Ec*LTAE) to synthesise unlabelled serine, representing a substrate for the production of unlabelled methylene-H_4_F by *Ec*GLYA [67]. Deletion of the respective genes is planned for follow-up experiments to improve efficiency of formate incorporation at low formate concentrations using C_1_S.

The highest total conversion achieved with C_1_S-*rganmt* was 46%, which has to be improved in order to use the strain on a larger scale. In future experiments, time courses showing the conversion of the MT substrate to the (^13^C-)methylated product in combination with analysis of the labelled and unlabelled C_1_-H_4_F metabolites, methionine, and SAM will be an important prerequisite to carry out flux analyses for identification of substantial bottlenecks. Based on this, overexpression or introduction of auxiliary pathways to improve product formation can be further explored.

The assimilation of formate, its reduction to methyl groups and the synthesis of SAM is an energy-consuming process, demanding two equivalents of ATP and two equivalents of NAD(P)H. Synthesis of homocysteine from aspartate further requires three equivalents of ATP and two equivalents of NADH. This increased demand for energy can stress *E. coli* and induce an imbalance in cofactor supply [27]. Increasing NADH regeneration by implementation of, *e.g.* formate dehydrogenase could improve the intracellular availability of reducing equivalents in *E. coli* and, as a result, also conversion [13].

## Conclusion

Our study demonstrates the introduction of the renewable C_1_-compound formate into SAM-derived methyl groups. Utilisation of pFCM in BL21-*fcm-rganmt-ecmetF* allowed methylation with 67% of ^13^C-labelled product, using the MTs *Pp*CaOMT and CouO more than 50% of introduced methyl groups were ^13^C-labelled. BL21-*fcm*-*mt* offers a simple opportunity to introduce formate’s carbon into value-added small molecules. Supply of formate to the engineered serine-dependent C_1_-auxotrophic *E. coli* strain C_1_S-*rganmt* successfully increased conversion by 57% with a share of 34% of ^13^C-labelled product; with this, formate can be used as a source for methyl groups. Starting from this, further bottlenecks of whole-cell methylation (*e.g.* time courses and flux analysis) can be investigated, and by the addition of auxiliary enzymes (*e.g.* formate dehydrogenase), and other optimisation steps (*e.g.* deletion of *ecpfl* or *ecltae*), it should be possible to further increase the performance of the strains. In summary, we describe a simple strategy on how to engineer *E. coli* to use the renewable C_1_-compound formate to support SAM-dependent methylation for future applications.

## Supplementary Information

Supplementary material 1.

## Data Availability

All data generated or analysed during this study are included in this published article and its supplementary information files. The datasets used and/or analysed during the current study are available from the corresponding author on reasonable request.
