# Supplementary material for: Rewiring Escherichia coli to transform formate into methyl groups
Source: Microb Cell Fact. 2025 Mar 7;24:55. doi: 10.1186/s12934-025-02674-4 (PMC11887345; doi:10.1186/s12934-025-02674-4)
Supplement: Supplementary file 1 — Supplementary material 1. [file 12934_2025_2674_MOESM1_ESM.pdf]

Supplementary information

Rewiring *Escherichia coli* to Transform Formate into Methyl Groups

Michael K. F. Mohr,[a] Ari Satanowski[b,c], Steffen N. Lindner [d], Tobias J. Erb[b,e], Jennifer N. Andexer[a]*

[a] Michael K. F. Mohr, Jennifer N. Andexer, Institute of Pharmaceutical Sciences, University of Freiburg, Albertstr. 25, 79104 Freiburg, Germany. jennifer.andexer@pharmazie.uni-freiburg.de

[b] Ari Satanowski, Tobias J. Erb, Max Planck Institute for Terrestrial Microbiology, Karl-von-Frisch-Straße 10, 35043 Marburg, Germany

[c] Ari Satanowski, Max Planck Institute of Molecular Plant Physiology, Am Mühlenberg 1, 14476 Potsdam-Golm, Germany

[d] Steffen N. Lindner-Mehlich, Department of Biochemistry, Charité Universitätsmedizin Berlin, corporate member of Freie Universität Berlin and Humboldt-Universität, Charitéplatz 1, 10117 Berlin, Germany

[e] Tobias J. Erb, LOEWE Center for Synthetic Microbiology (SYNMIKRO), Philipps University of Marburg, Marburg, Germany

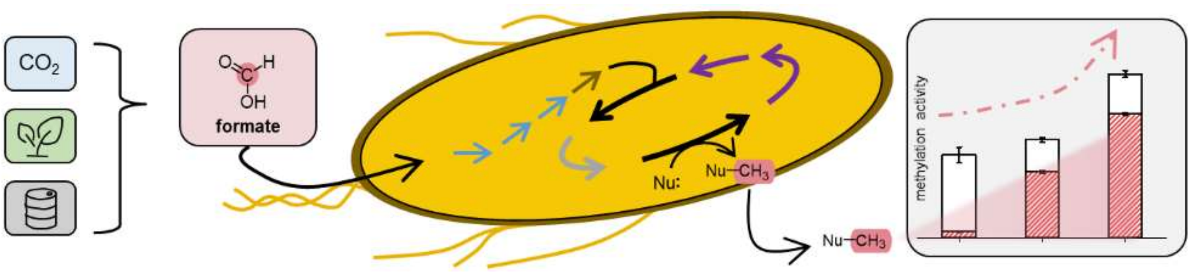

Supplementary figures & data

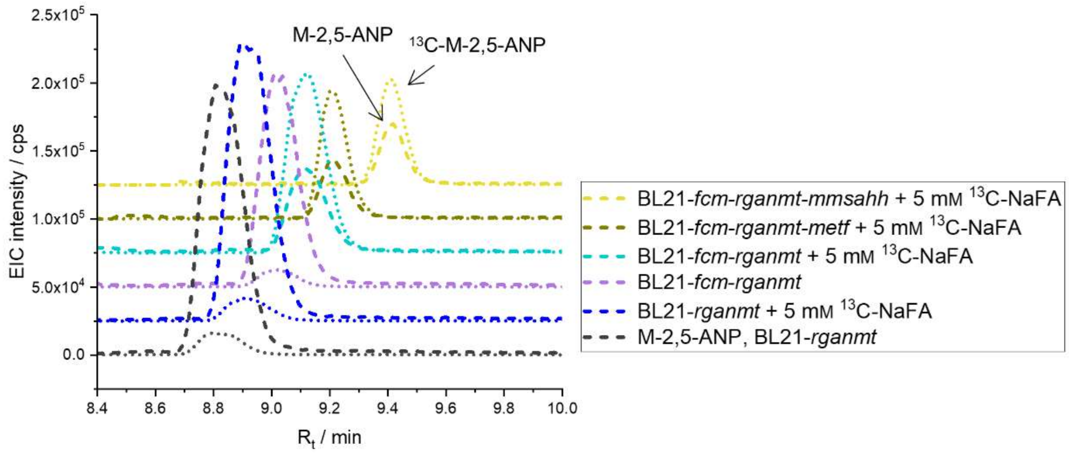

Figure SI 1. Analysis of formate-derived methyl groups with BL21-*fcm-rganmt*. Shown is the LC-MS/MS analysis of ^{13}C-labelled product with BL21-*fcm-rganmt* and strains co-overexpressing genes from the SAM regeneration cycle or *ecmetF*, when 5 mM ^{13}C-formate was supplied. Experiments were conducted in M9-medium (22 mM glucose) with a final OD_{600} of 3.0, 0.75 mM of substrate to be methylated, 0 - 5 mM ^{13}C-formate at 37 °C, 170 rpm for 24 h. Experiments were conducted in biological triplicates.

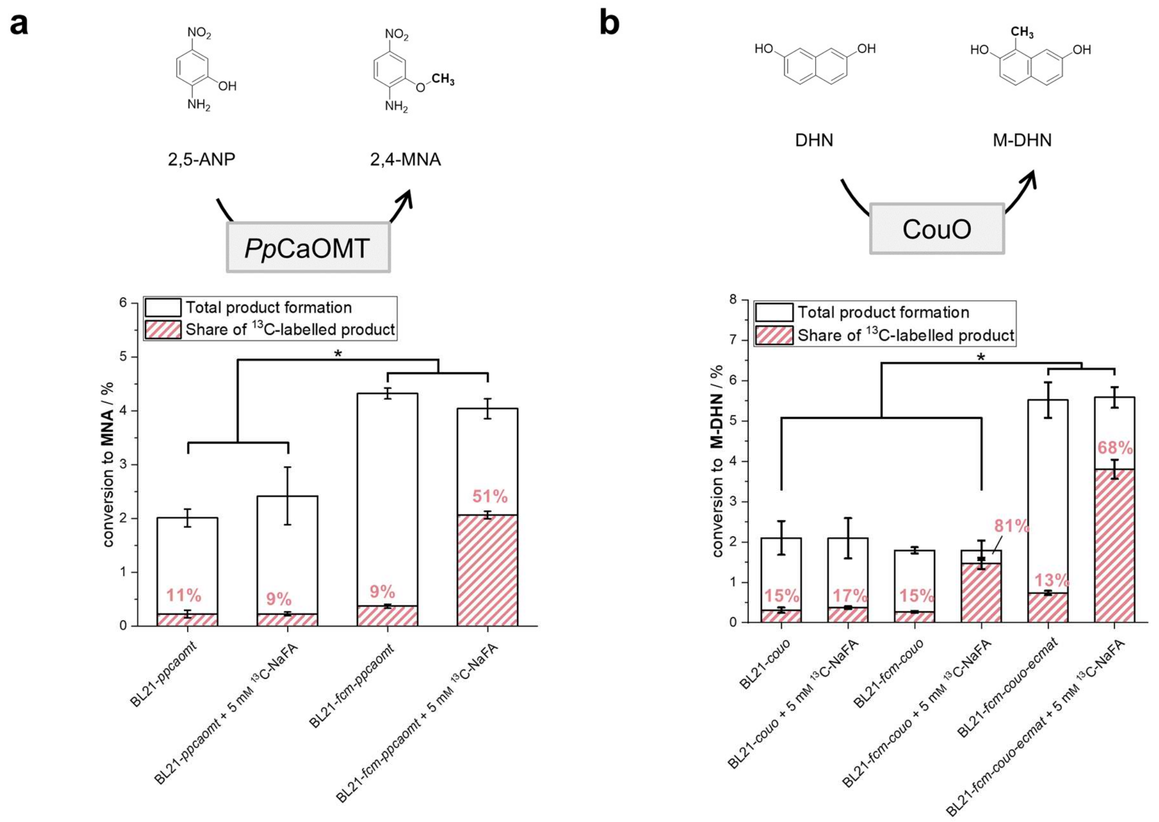

Figure SI 2. General applicability of methylation from formate with BL21-*fcm*. Bar charts show production of methylated products with BL21-*ppcaomt* (**a**) or BL21-*couo* (**b**) with and without co-expression of *fcm* and supply with 5 mM ^{13}C-formate. Experiments were conducted in M9-medium (22 mM glucose) with a final OD_{600} of 3.0, 0.75 mM of substrate to be methylated, 0 - 5 mM ^{13}C-formate at 37 °C, 170 rpm for 24 h. Experiments were conducted in biological triplicates.

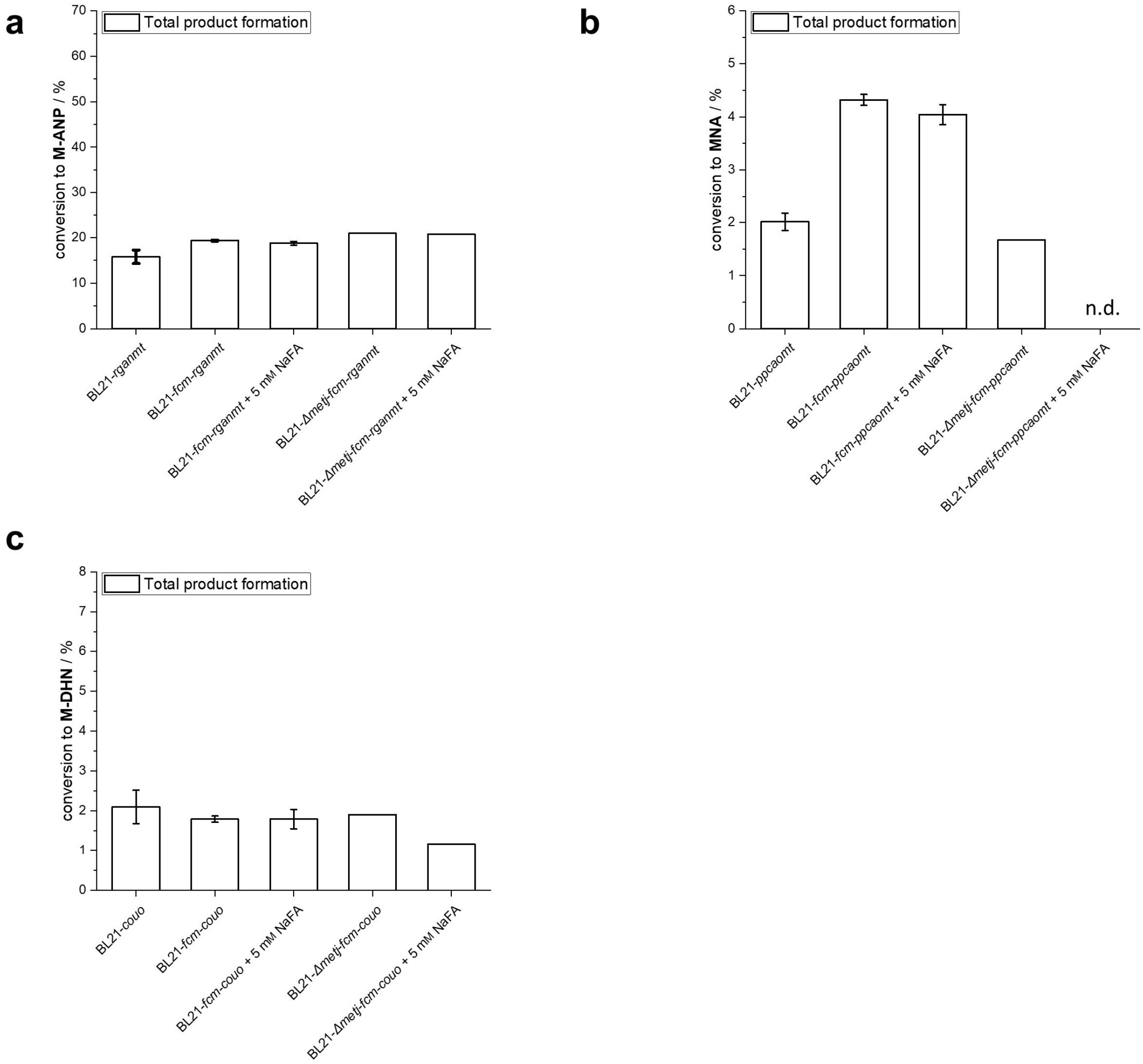

Figure SI 3. Comparison of *in vivo* methylation from formate with BL21*::Δmetj-fcm* and BL21-*fcm*. Shown is product formation of the strains bearing **a** *Rg*ANMT, **b** *Pp*CaOMT and **c** CouO in BL21, BL21-*fcm* and BL21*::Δmetj-fcm* with and without the addition of formate. Experiments were conducted in M9-medium (22 mM glucose) with a final OD_{600} of 3.0, 0.75 mM of substrate to be methylated, 0 - 5 mM ^{13}C-formate at 37 °C, 170 rpm for 24 h. NOTE: In this experiment no ^{13}C-labelled formate was used. Experiments were performed in biological triplicates. n. d. = not detected.

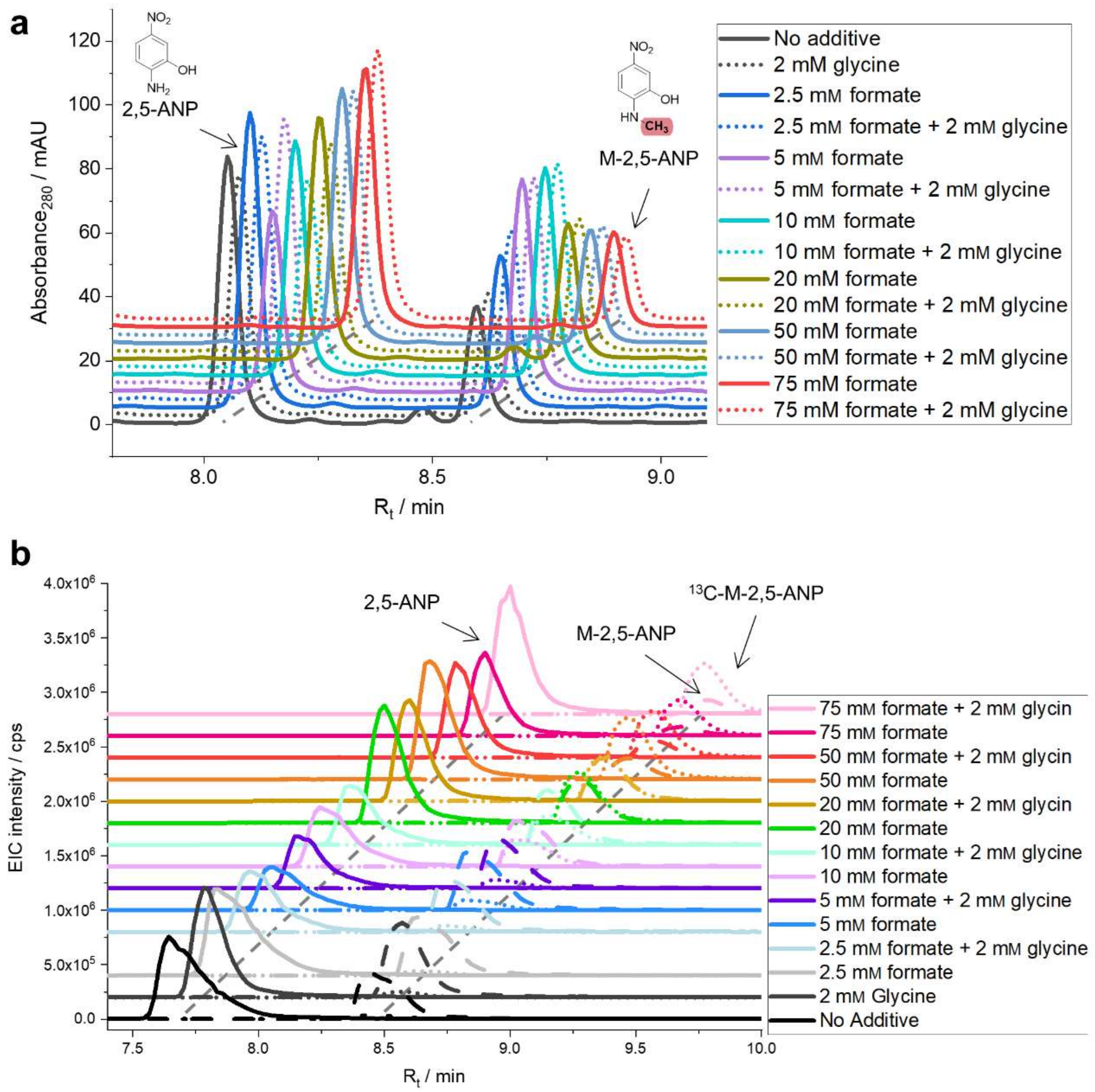

Figure SI 4. Screening of different ^{13}C-formate concentrations with C_1S-*rganmt*. a Production of M-2,5-ANP was analysed with HPLC-UV (λ = 280 nm) using C_1S-*rganmt* with formate concentrations ranging from 2.5 to 75 mM ^{13}C-formate with and without the addition of 2 mM glycine. **b** LC-MS/MS analysis of the incorporation of formate-derived methyl groups in M-2,5-ANP. Experiments were conducted in M9-medium (22 mM glucose) with a final OD_{600} of 3.0, 0.75 mM ANP, 2.5 - 75 mM ^{13}C-formate with or without 2 mM glycine at 37 °C, 170 rpm for 24 h in biological triplicates.

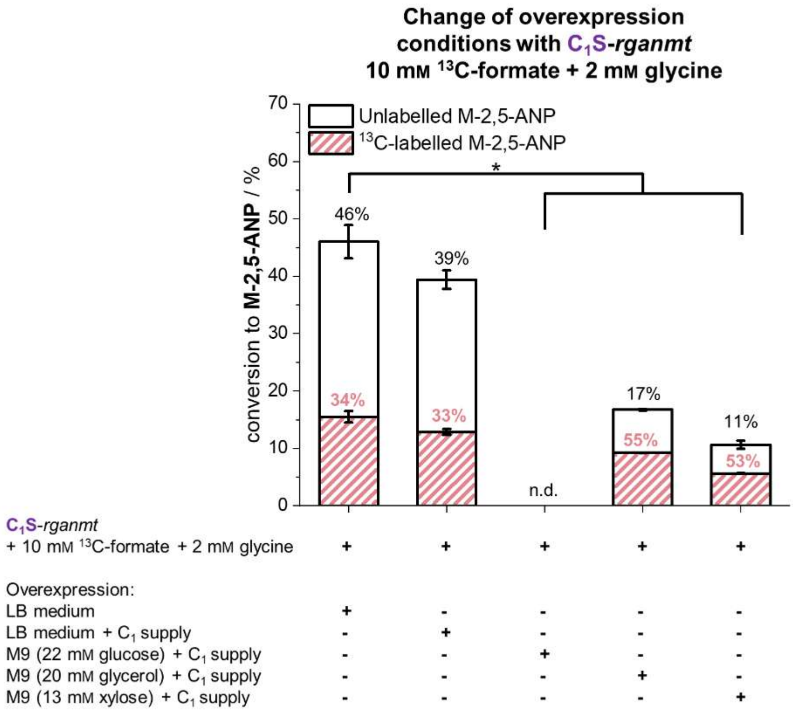

Figure SI 5. Exchange of overexpression conditions for C_1S-*rganmt*. It was suspected that the low share of ^{13}C-labelled product would derive from a carryover or residual free intracellular methionine from the overexpression phase in LB-medium. Therefore, overexpression as well as biotransformation conditions were altered to: overexpression in LB-medium with formate and glycine supply or to M9 medium supplied with 22 mM glucose, 20 mM glycerol, or 13 mM xylose. In this figure, the conversion of 2,5-ANP to M-2,5-ANP by C_1S-*rganmt* (black) and the share of ^{13}C-labelled product (red) are given. Conditions during overexpression and biotransformation phase were altered as indicated to M9 (22 mM glucose, 20 mM glycerol, or 13 mM xylose) at a final OD_{600} of 3.0, 0.75 mM 2,5-ANP, 10 mM ^{13}C-formate and 2 mM glycine at 37 °C, 170 rpm for 24 h. n.d. = not detected.

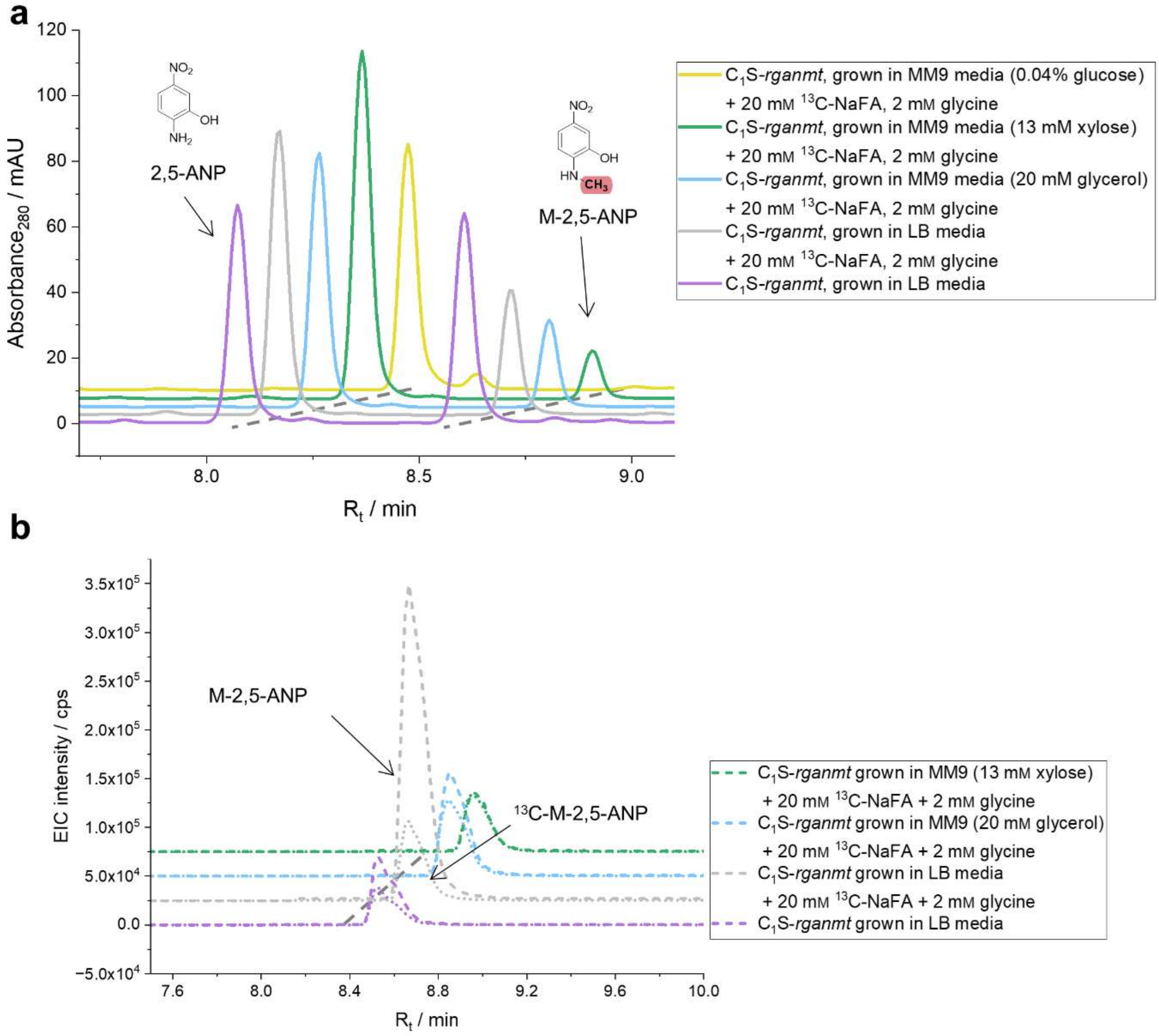

Figure SI 6. Media screening to increase methylation from formate with C_1S-*rganmt*. a Total methylation of 2,5-ANP to M-2,5-ANP through C_1S-*rganmt* grown and overexpressed in different medium was analysed with HPLC-UV (λ = 280 nm). **b** The share of labelled product was analysed *via* LC-MS/MS. Striped lines represent M-2,5-ANP (m/z = 169.050-123.050) and dotted lines represent ^{13}C-M-2,5-ANP (m/z = 170.050-124.050). Cells were grown and overexpressed in the respective medium and the experiments were conducted in M9-medium (22 mM glucose or with the respective M9-medium the cultures were grown in) with a final OD_{600} of 3.0, 0.75 mM 2,5-ANP, 10 mM ^{13}C-formate with 2 mM glycine at 37 °C, 170 rpm for 24 h in biological triplicates.

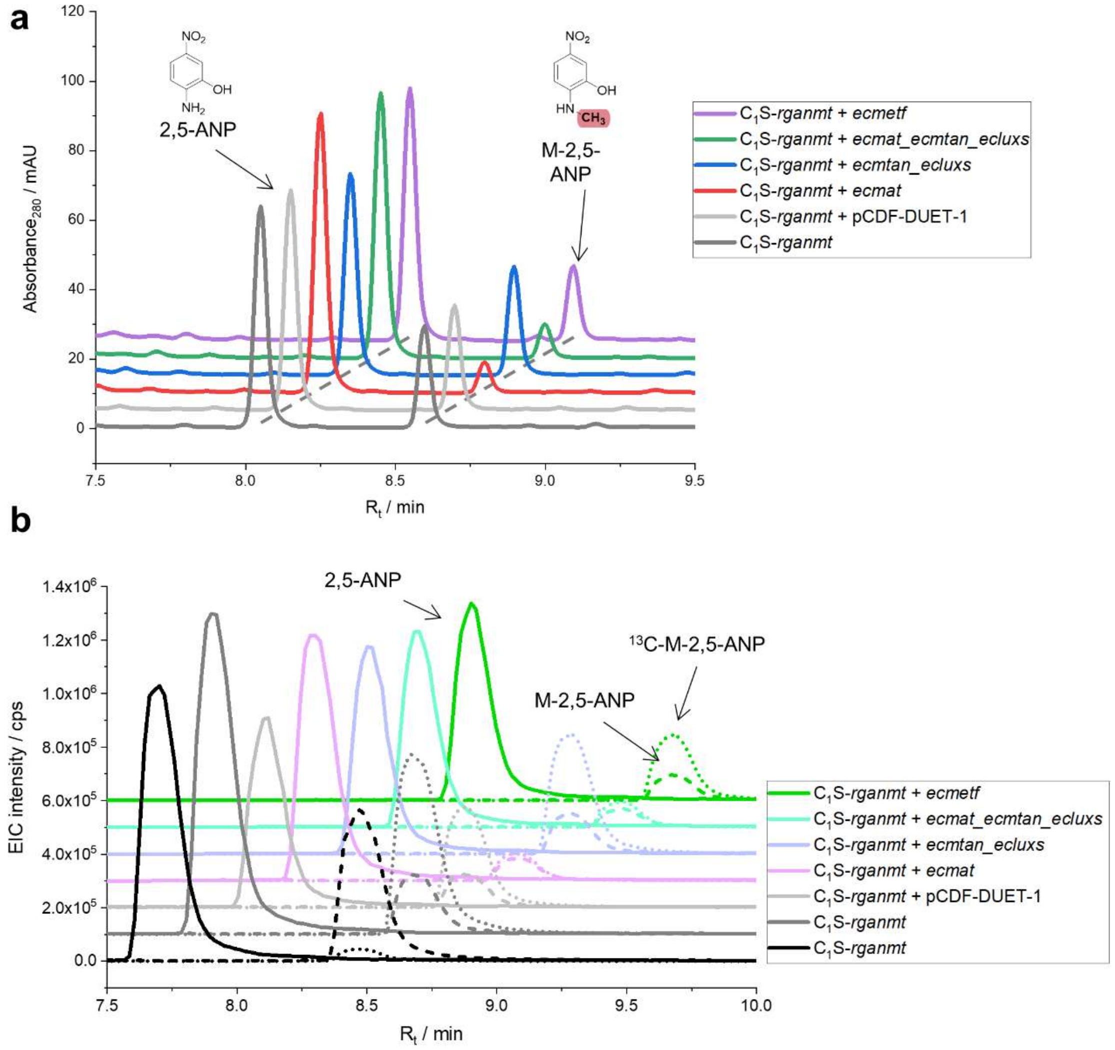

Figure SI 7. Co-overexpression of SAM regeneration cycle genes in C_1S-*rganmt*. a Production of M-2,5-ANP was analysed with HPLC-UV (λ = 280 nm) with C_1S-*rganmt* in combination with the SAM regeneration cycle genes encoded on a pCDF--DUET-1 vector using 50 mM ^{13}C-formate. **b** Incorporation of ^{13}C-formate was analysed with LC-MS/MS. Compact lines represents 2,5-ANP (m/z = 155.050-109.050), striped lines represent M-2,5-ANP (m/z = 169.050-123.050) and dotted lines represent ^{13}C-M-2,5-ANP (m/z = 170.050-124.050). Experiments were conducted in M9-medium (22 mM glucose) with a final OD_{600} of 3.0, 0.75 mM 2,5-ANP, and 50 mM ^{13}C-formate at 37 °C, 170 rpm for 24 h in biological triplicates.

Table SI 1. Production of M-2,5-ANP for different strains and conditions.

Strain	Condition	Conversion ± SD [%]	Fold change ± SD	Share of ^{13}C-labelled M-2,5-ANP [%]
BL21-*rganmt*	-	15.8 ± 1.5	1 ± 0.09	7.7 ± 0.4
	5 mM ^{13}C-formate	15.5 ± 0.2	0.98 ± 0.01	7.8 ± 0.2
BL21-*fcm-rganmt*	-	19.3 ± 0.1	1.22 ± 0.01	7.6 ± 0.3
	5 mM ^{13}C-formate	18.7 ± 0.4	1.19 ± 0.03	67.3 ± 1.4
BL21-*fcm-rganmt-ecmat*	5 mM ^{13}C-formate	-	-	-
BL21-*fcm-rganmt-mmsahh*	5 mM ^{13}C-formate	17.4 ± 1.3	1.10 ± 0.09	64.1 ± 1.5
BL21-*fcm-rganmt-ecmetF*	5 mM ^{13}C-formate	20.5 ± 0.8	1.30 ± 0.05	66.6 ± 1.8
C_1-opt-*rganmt*	-	2.2 ± 0.15	0.14 ± 0.01	9.1 ± 1.2
	5 mM ^{13}C-formate	5.1 ± 0.15	0.32 ± 0.01	53.6 ± 0.96
C_1S-*rganmt*	-	29.3 ± 1.8	1.9 ± 0.1	8.0 ± 0.3
	2.5 mM formate	34.4 ± 1.0	2.2 ± 0.1	11.7 ± 0.4
	5 mM formate	41.8 ± 6.4	2.6 ± 0.4	15.0 ± 1.4
	10 mM formate	43.7 ± 2.8	2.8 ± 0.2	37.8 ± 1.4
	20 mM formate	38.0 ± 2.9	2.4 ± 0.2	56.0 ± 1.4
	50 mM formate	31.2 ± 0.6	2.0 ± 0.1	75.7 ± 0.7
	75 mM formate	27.0 ± 1.6	1.7 ± 0.1	80.8 ± 0.4
	2 mM glycine	32.3 ± 1.7	2.0 ± 0.1	10.5 ± 4.1
	2.5 mM formate + 2 mM glycine	38.9 ± 3.2	2.5 ± 0.2	11.1 ± 0.2
	5 mM formate + 2 mM glycine	44.2 ± 0.3	2.8 ± 0.1	15.5 ± 0.4
	10 mM formate + 2 mM glycine	46.0 ± 4.2	2.9 ± 0.3	33.7 ± 2.2
	20 mM formate + 2 mM glycine	40.0 ± 2.6	2.5 ± 0.2	54.3 ± 2.2
	50 mM formate + 2 mM glycine	30.4 ± 1.0	1.9 ± 0.1	73.5 ± 1.5
	75 mM formate + 2 mM glycine	24.3 ± 1.5	1.5 ± 0.1	80.3 ± 1.15
C_1S-*rganmt*	Overexpression in LB-medium + 20 mM ^{13}C-formate + 2 mM glycine, assay in M9 (22 mM glucose); 10 mM ^{13}C-formate + 2 mM glycine	39.4 ± 1.6	2.49 ± 0.10	32.7 ± 1.28
C_1S-*rganmt*	Overexpression in M9 (22 mM glucose) + 20 mM ^{13}C-formate + 2 mM glycine, assay in M9 (22 mM glucose) 10 mM ^{13}C-formate + 2 mM glycine	-	-	-
C_1S-*rganmt*	Overexpression in M9 (20 mM glycerol) + 20 mM ^{13}C-formate + 2 mM glycine, assay in M9 (20 mM glycerol) 10 mM ^{13}C-formate + 2 mM glycine	16.8	1.06 ± 0.01	55.0 ± 0.18

C_1S-*rganmt*	Overexpression in M9 (13 mM xylose) + 20 mM ^{13}C-formate + 2 mM glycine, assay in M9 (13 mM xylose) 10 mM ^{13}C-formate + 2 mM glycine	10.6 ± 0.7	0.67 ± 0.04	53.2 ± 0.6
C_1S-*rganmt-empty*	50 mM ^{13}C-formate	31.5 ± 0.9	2.0 ± 0.1	76.6 ± 0.8
C_1S-*rganmt-ecmat*	50 mM ^{13}C-formate	10.5 ± 1.0	0.7 ± 0.1	57.3 ± 1.1
C_1S-*rganmt-ecmtan-ecluxS*	50 mM ^{13}C-formate	33.6 ± 0.6	2.1 ± 0.1	76.9 ± 0.6
C_1S-*rganmt-ecmat-ecmtan-ecluxS*	50 mM ^{13}C-formate	11.3 ± 0.2	0.7 ± 0.1	60.6 ± 1.6
C_1S-*rganmt-ecmetF*	50 mM ^{13}C-formate	23.5 ± 1.3	1.5 ± 0.1	74.0 ± 1.1

HPLC-UV analysis

HPLC-UV analysis was used to detect MT products generated during *in vivo* methylation.

Table SI 2. HPLC-UV analysis for quantification of methylated products.

	HPLC-UV analysis for quantification of M-ANP
HPLC system	Agilent 1260 infinity II
Column	ISAspher 100-3-C18 (150 x 4.0 mm)
Mobile phase A (aqueous)	0.1% formic acid
Mobile phase B (organic)	acetonitrile
Detection wavelength	280 nm
Flow rate	1 ml/ min
Sample volume injected	10 µl
Equilibration	97% A
Gradient	4 min 97% A/ 3% B
	3 min to 30% A/ 70% B
	0.1 min to 100% B
	2.9 min 100% B
	1 min to 97% A/ 3% B
	3 min 97% A/ 3% B

LC-MS/MS analysis

LC-MS/MS was used for analysis of the share of ^{13}C-labelled products.

Table SI 3. LC-MS/MS method for determination of ^{13}C-labelled products.

	LC-MS/MS method for determination of ^{13}C-labelled M-ANP
LC-MS system	SCIEX 5500+
Column	ISAspher 120-3-C18 Aq (50 x 2.1 mm)
Mobile phase A (aqueous)	0.1% formic acid
Mobile phase B (organic)	acetonitril
Flow rate	0.2 ml/ min
MS settings	Curtain Gas: 25 psi
	Ion spray voltage: +2900 eV
	Temperature: 450 °C
	Ion source gas 1: 50 psi
	Ion source gas 2: 50 psi
	Declustering potential: 10 eV
	Exit potential: 12 eV
Sample preparation	Dilution 1:10 and filtration
Sample volume injected	1 µl
Equilibration	97% A
Gradient	4 min 97% A/ 3% B
	3 min to 30% A/ 70% B
	0.1 min to 100% B
	2.9 min 100% B
	1 min to 97% A/ 3% B
	3 min 97% A/ 3% B

Table SI 4. Multiple reaction monitoring transitions for LC-MS/MS measurements.

Substrate	Q1 mass	Q3 mass
2,5-ANP (2,5-aminonitrophenol)	155.050	155.050
	155.050	109.050
M-ANP (*N*-methyl-2,5-aminonitrophenol)	169.050	169.050
	169.050	123.050
^{13}C-M-ANP	170.050	170.050
(*N*-methyl-2,5-aminonitrophenol)	170.050	124.050
2,4-MNA	169.050	169.050
(4-methoxy-2-nitroaniline)	169.050	123.050
^{13}C-2,4-MNA	170.050	170.050
	170.050	124.050
DHN (2,7-dihydroxynaphthalene)	161.050	161.050
Methyl-DHN (1-methyl-2,7-dihydroxynaphthalene)	175.070	175.070
^{13}C-Methyl-DHN	176.070	176.070

Gene sequences used in this study:

mexftfl (Formate-H_4F ligase from *Methylorubrum extorquens* AM1)

ATGCCGAGCGATATTGAAATTGCCCGTGCAGCAACCCTGAAACCGATTGCACAGGTTGCAGAAAAACTGGGTATTCCGGATGAAGCAC
TGCATAACTATGGTAAACATATCGCCAAAATCGACCACGATTTCATTGCAAGCCTGGAAGGTAAACCGGAAGGCAAACTGGTTCTGGT
TACCGCAATTAGCCCGACACCGGCAGGCGAAGGTAAAACCACCACCACCGTTGGTCTGGGTGATGCACTGAATCGTATTGGTAAACGT
GCAGTTATGTGTCTGCGTGAACCGAGCCTGGGTCCGTGTTTTGGTATGAAAGGTGGTGCAGCAGGCGGTGGTAAAGCCCAGGTTGTGC
CGATGGAACAAATCAATCTGCATTTTACCGGTGATTTTCATGCAATTACCAGCGCACATAGCCTGGCAGCAGCACTGATTGATAATCA
TATCTATTGGGCCAACGAGCTGAATATTGATGTTCGTCGTATTCATTGGCGTCGTGTTGTTGATATGAATGATCGTGCACTGCGTGCA
ATTAATCAGAGCTTAGGTGGTGTTGCAAATGGTTTTCCGCGTGAAGATGGTTTTGATATCACCGTTGCAAGCGAAGTTATGGCAGTGT
TTTGTCTGGCCAAAAATCTGGCAGATCTGGAAGAACGTCTGGGTCGTATTGTTATTGCAGAAACCCGTGATCGTAAACCGGTTACACT
GGCAGATGTTAAAGCAACCGGTGCCATGACCGTTCTGCTGAAAGATGCACTGCAGCCGAATCTGGTTCAGACCCTGGAAGGCAATCCG
GCACTGATTCATGGTGGTCCGTTTGCAAATATTGCCCATGGTTGTAATAGCGTTATTGCAACCCGTACCGGTCTGCGTCTGGCCGATT
ATACCGTTACCGAAGCAGGTTTTGGTGCCGATCTGGGTGCCGAAAAATTCATTGACATTAAATGTCGTCAGACGGGTCTGAAACCGTC
AGCAGTTGTTATTGTTGCAACCATTCGTGCCCTGAAAATGCATGGTGGTGTTAATAAGAAAGATCTGCAGGCAGAAAATCTGGATGCA
CTGGAAAAAGGTTTTGCAAATCTGGAACGCCATGTGAATAATGTTCGTAGCTTTGGTCTGCCGGTTGTTGTTGGTGTGAATCATTTCT
TTCAGGATACCGATGCAGAACATGCACGTCTGAAAGAACTGTGTCGTGATCGTCTGCAGGTTGAAGCAATTACCTGTAAACATTGGGC
TGAAGGCGGTGCCGGTGCAGAAGCACTGGCACAGGCCGTTGTTAAACTGGCCGAAGGTGAACAGAAACCGCTGACCTTTGCATATGAA
ACCGAAACCAAAATTACCGACAAAATCAAAGCCATTGCCACCAAACTGTATGGTGCAGCCGATATTCAGATTGAAAGCAAAGCAGCAA
CCAAACTGGCAGGCTTTGAAAAAGATGGTTATGGCAAACTGCCGGTTTGTATGGCAAAAACCCAGTATAGCTTTAGCACCGATCCGAC
ACTGATGGGTGCACCGAGCGGTCATCTGGTTAGCGTTCGTGATGTGCGTCTGAGCGCAGGCGCTGGTTTTGTTGTTGTTATTTGTGGT
GAAATCATGACCATGCCAGGTCTGCCGAAAGTTCCGGCAGCAGATACCATTCGTCTGGATGCCAATGGTCAGATTGATGGTCTGTTTT
AA

mexfch (Formyl-H_4F cyclohydrolase from *Methylorubrum extorquens* AM1)

ATGGCCGGCAACGAGACGATCGAAACATTCCTCGATGGCCTGGCGAGCTCGGCCCCGACCCCCGGCGGCGGCGGTGCCGCCGCGATCT
CCGGCGCCATGGGCGCGGCGCTGGTCTCGATGGTGTGTAACCTCACCATCGGCAAGAAGAAGTATGTCGAGGTCGAGGCCGACCTGAA
GCAGGTGCTGGAGAAGTCGGAAGGCCTGCGCCGCACGCTCACCGGCATGATCGCCGACGACGTCGAGGCTTTCGACGCGGTGATGGGC
GCCTACGGGCTGCCGAAAAACACCGACGAGGAGAAGGCTGCCCGCGCCGCCAAGATTCAGGAGGCGCTCAAGACCGCGACCGACGTGC
CGCTCGCCTGCTGCCGCGTCTGCCGCGAGGTGATCGATCTGGCCGAGATCGTCGCCGAGAAGGGCAATCTCAACGTCATCTCGGATGC
CGGCGTCGCCGTGCTCTCGGCCTATGCCGGTCTGCGCTCGGCGGCCCTCAACGTCTACGTCAACGCCAAGGGCCTCGACGACCGCGCC
TTCGCCGAGGAGCGGCTGAAGGAGCTGGAAGGCCTGCTGGCCGAGGCGGGCGCGCTCAACGAGCGGATCTACGAGACGGTGAAGTCCA
AGGTAAACTGA

mexmtdA (Methylene-H_4F dehydrogenase A from *Methylorubrum extorquens* AM1)

ATGTCCAAGAAGCTGCTCTTCCAGTTCGACACCGATGCCACGCCGAGCGTCTTCGACGTCGTCGTCGGCTACGACGGCGGTGCCGACC
ACATCACCGGCTACGGCAACGTCACGCCCGACAACGTCGGCGCCTATGTCGACGGCACGATCTACACCCGCGGCGGCAAGGAGAAGCA
GTCGACGGCGATCTTCGTCGGCGGCGGCGACATGGCGGCCGGCGAGCGGGTGTTCGAGGCGGTGAAGAAGCGCTTCTTCGGCCCGTTC
CGCGTGTCCTGCATGCTGGATTCGAACGGCTCCAACACGACCGCCGCGGCGGGTGTGGCGCTCGTCGTCAAGGCGGCGGGCGGCTCGG
TCAAGGGCAAGAAGGCCGTCGTGCTCGCGGGCACCGGCCCGGTCGGCATGCGCTCGGCGGCGCTGCTCGCCGGCGAGGGCGCCGAGGT
CGTGCTGTGCGGGCGCAAGCTCGACAAGGCGCAGGCCGCGGCCGATTCCGTGAACAAGCGCTTCAAGGTGAACGTCACCGCGGCCGAA
ACCGCGGACGACGCTTCGCGCGCCGAGGCCGTGAAGGGCGCCCATTTCGTCTTCACCGCCGGTGCGATCGGCCTTGAACTGCTGCCGC
AGGCAGCCTGGCAGAACGAGAGTTCGATCGAGATCGTGGCCGACTACAACGCCCAGCCGCCGCTCGGCATCGGCGGGATCGATGCGAC
CGACAAAGGCAAGGAATACGGCGGAAAGCGCGCCTTCGGTGCGCTCGGCATCGGCGGCTTGAAGCTCAAGCTGCACCGCGCCTGCATC
GCCAAGCTGTTCGAGTCGAGCGAAGGCGTCTTCGACGCCGAGGAGATCTACAAGCTGGCCAAGGAAATGGCCTGA

rganmt (His_6-tagged, anthranilate *N*-MT from *Ruta graveolens*)

ATGGGCAGCAGCCATCATCATCATCATCACAGCAGCGGCCTGGTGCCGCGCGGCAGCCATATGGGTAGCCTGAGCGAAAGCCATACCC
AGTATAAACATGGTGTTGAAGTGGAAGAAGATGAAGAGGAATCATATAGCCGTGCAATGCAGCTGAGCATGGCAATTGTTCTGCCGAT
GGCAACCCAGAGCGCAATTCAGCTGGGTGTTTTTGAAATTATTGCAAAAGCACCGGGTGGTCGTCTGAGCGCAAGCGAAATTGCAACC
ATTCTGCAGGCACAGAATCCGAAAGCTCCGGTTATGCTGGATCGTATGCTGCGTCTGCTGGTTAGCCATCGTGTTCTGGATTGTAGCG
TTAGCGGTCCGGCAGGCGAACGTCTGTATGGTCTGACCAGCGTTAGCAAATATTTCGTTCCGGATCAGGATGGTGCAAGCCTGGGCAA
TTTTATGGCACTGCCGCTGGATAAAGTTTTTATGGAAAGCTGGATGGGTGTTAAAGGTGCAGTTATGGAAGGTGGTATTCCGTTTAAT
CGTGTTCATGGCATGCACATCTTTGAATATGCAAGCAGCAATAGCAAATTCAGCGATACCTATCATCGTGCCATGTTTAATCATAGCA
CCATTGCACTGAAACGCATCCTGGAACACTATAAAGGTTTTGAGAATGTGACCAAACTGGTTGATGTTGGTGGTGGTCTGGGTGTTAC
CCTGAGCATGATTGCAAGCAAATATCCGCATATTCAGGCCATCAATTTTGATCTGCCGCATGTTGTTCAGGATGCAGCAAGCTATCCG
GGTGTTGAACATGTGGGTGGTAATATGTTTGAAAGCGTTCCGGAAGGTGATGCCATTCTGATGAAATGGATTCTGCATTGTTGGGATG
ATGAACAGTGTCTGCGTATTCTGAAAAACTGCTATAAAGCCACACCGGAAAATGGCAAAGTGATTGTTATGAATAGCGTTGTTCCGGA
AACACCGGAAGTTAGCAGCAGCGCACGTGAAACCAGCCTGCTGGATGTTCTGCTGATGACCCGTGATGGTGGTGGCCGTGAACGTACC
CAGAAAGAATTTACCGAACTGGCAATTGGTGCAGGTTTTAAAGGTATTAACTTTGCCTGTTGCGTGTGCAACCTGCATATTATGGAAT
TTTTCAAATAA

ppcaomt (His$_6$-tagged, caffeate *O*-MT form *Prunus persica*)

ATGGGCAGCAGCCATCATCATCATCATCACAGCAGCGGCCTGGTGCCGCGCGGCAGCCATATGGCAAGCAGCCTGGAACGTAAAAGCC
ATCCGAAAATTAACCATGCAGAACCGGAAGATGAAATCACCAAAGAAGAAGAGGACGAGAGCTTTTGTTATGCAATGCAGCTGGTTGG
TAGCAGCGTTCTGAGCATGAGCCTGCAGAGCGCAATTAAACTGGGTATCTTTGATATCATTGCACGTAAAGGTCCGGGTGCAAAACTG
AGCAGCAGCGAAATTGCAACCAAAATTGGCACCGAAAATCCGGAAGCACCGGTTATGGTTGATCGTATTCTGCGTCTGCTGACCAGCC
ATAGCGTGCTGAATTGTAGCGCAGTTGCAGCAAATGGTGGTAGCGATTTTCAGCGTGTTTATAGCCTGGGTCCTGTTAGCAAATATTT
CGTGAATGATGAAGAAGGTGGTAGTCTGGGTCCGCTGCTGACCCTGATTCAGGATCGTGTTTTTCTGGAAAGCTGGTCACAGCTGAAA
GATGCAGTTGTTGAAGGTGGCATTCCGTTTAATCGTGTTCATGGTATGCATGCCTTTGAATATCCTGGTCTGGATCCGCGTTTTAATC
AGGTGTTTAATACCGCCATGTTTAACCATACAACCATCGTGATCAAAAAGCTGCTGCATATCTATAAAGGCCTGGAAGATAAAAATCT
GACCCAGCTGGTAGATGTTGGTGGTGGCCTGGGTGTTACCCTGAATCTGATTACCAGCCGTTATCAGCATATCAAGGGCATTAACTTT
GATCTGCCGCATGTTGTTAATCATGCACCGAGCTATCCGGGTGTTGAACATGTTGGCGGTGATATGTTTGCAAGCGTTCCGAGCGGTG
ATGCCATTTTTATGAAATGGATTCTGCATGATTGGAGCGACGAACATTGTCTGAAACTGCTGAAAAATTGCTATAAAGCGATTCCGGA
TAACGGCAAAGTTATTGTTGTGGAAGCACTGCTGCCTGCAATGCCGGAAACCAGCACCGCCACCAAAACCACCAGTCAGCTGGATGTT
CTGATGATGACCCAGAATCCTGGTGGTAAAGAACGTAGCGAACAAGAATTTATGGCACTGGCAACCGGTGCAGGTTTTAGCGGTATTC
GTTATGAATGCTTTGTGTGCAATTTCTGGGTGATGGAATTTTTCAAATAA

couO (His$_6$-tagged, aminocoumarin *C*-MT from *Steptromyces rishiriensis*)

ATGGGCAGCAGCCATCATCATCATCATCACAGCAGCGGCCTGGTGCCGCGCGGCAGCCATATGGCTGCCGCGCGGCACCAGGCCGCTG
CTGTGATGATGATGATGATGGCTGCTGCCCATATGAAAATTGAACCGATTACCGGCAGCGAAGCGGAAGCGTTTCATCGCATGGGCAG
CCGCGCGTTTGAACGCTATAACGAATTTGTGGATCTGCTGGTGGGCGCGGGCATTGCGGATGGCCAGACCGTGGTGGATCTGTGCTGC
GGCAGCGGCGAACTGGAAATTATTCTGACCAGCCGCTTTCCGAGCCTGAACCTGGTGGGCGTGGATCTGAGCGAAGATATGGTGCGCA
TTGCGCGCGATTATGCGGCGGAACAGGGCAAAGAACTGGAATTTCGCCATGGCGATGCGCAGAGCCCGGCGGGCATGGAAGATCTGCT
GGGCAAAGCGGATCTGGTGGTGAGCCGCCATGCGTTTCATCGCCTGACCCGCCTGCCGGCGGGCTTTGATACCATGCTGCGCCTGGTG
AAACCGGGCGGCGCGATTCTGAACGTGAGCTTTCTGCATCTGAGCGATTTTGATGAACCGGGCTTTCGCACCTGGGTGCGCTTTCTGA
AAGAACGCCCGTGGGATGCGGAAATGCAGGTGGCGTGGGCGCTGGCGCATTATTATGCGCCGCGCCTGCAGGATTATCGCGATGCGCT
GGCGCAGGCGGCGGATGAAACCCCGGTGAGCGAACAGCGCATTTGGGTGGATGATCAGGGCTATGGCGTGGCGACCGTGAAATGCTTT
GCGCGCCGCGCGGCGGCG

ecmat (His$_6$-tagged, methionine adenosyltransferase from *Escherichia coli*)

ATGGCAAAACACCTTTTTACGTCCGAGTCCGTCTCTGAAGGGCATCCTGACAAAATTGCTGACCAAATCTCTGATGCCGTTTTAGACG
CGATCCTCGAACAGGATCCGAAAGCACGCGTTGCTTGCGAAACCTACGTAAAAACCGGCATGGTTTTAGTTGGCGGCGAAATCACCAC
CAGCGCCTGGGTAGACATCGAAGAGATCACCCGTAACACCGTTCGCGAAATTGGCTATGTGCATTCCGACATGGGCTTTGACGCTAAC
TCCTGTGCAGTTCTGAGCGCTATCGGCAAACAGTCTCCTGACATCAACCAGGGCGTTGACCGTGCCGATCCGCTGGAACAGGGCGCGG
GTGACCAGGGTCTGATGTTTGGCTACGCAACTAATGAAACCGACGTGCTGATGCCAGCACCTATCACCTATGCACACCGTCTGGTACA
GCGTCAGGCTGAAGTGCGTAAAAACGGCACTCTGCCGTGGCTGCGCCCGGACGCGAAAAGCCAGGTGACTTTCCAGTATGACGACGGC
AAAATCGTTGGTATCGATGCTGTCGTGCTTTCCACTCAGCACTCTGAAGAGATCGACCAGAAATCGCTGCAAGAAGCGGTAATGGAAG
AGATCATCAAGCCGATTCTGCCCGCTGAATGGCTGACTTCTGCCACCAAATTCTTCATCAACCCGACCGGTCGTTTTGTTATCGGTGG
CCCGATGGGTGACTGCGGTCTGACTGGTCGTAAAATTATCGTTGATACCTACGGCGGCATGGCGCGTCACGGTGGCGGTGCATTCTCT
GGTAAAGATCCATCAAAAGTGGACCGTTCCGCAGCCTACGCAGCACGTTATGTCGCGAAAAACATCGTTGCTGCTGGCCTGGCCGATC
GTTGTGAAATTCAGGTTTCCTACGCAATCGGCGTGGCTGAACCGACTTCCATCATGGTAGAAACTTTCGGTACTGAGAAAGTGCCTTC
TGAACAACTGACTCTGCTGGTACGTGAGTTCTTCGACCTGCGCCCATACGGTCTGATTCAGATGCTGGATCTGCTGCACCCGATCTAC
AAAGAAACCGCAGCATACGGTCACTTTGGTCGTGAACATTTCCCGTGGGAAAAAACCGACAAAGCGCAGCTGCTGCGCGATGCTGCCG
GTCTGAAGTAA

ecmtan (His$_6$-tagged, *S*-methyl-5´-thioadenosine/ SAH nucleosidase from *Escherichia coli*)

ATGGCTGCCGCGCGGCACCAGGCCGCTGCTGTGATGATGATGATGATGGCTGCTGCCCATATGAAAATCGGCATCATTGGTGCAATGG
AAGAAGAAGTTACGCTGCTGCGTGACAAAATCGAAAACCGTCAAACTATCAGTCTCGGCGGTTGCGAAATCTATACCGGCCAACTGAA
TGGAACCGAGGTTGCGCTTCTGAAATCGGGCATGGTAAAGTCGCTGCGGCGCTGGGTGCCACTTTGCTGTTGGAACACTGCAAGCCAG
ATGTGATTATTAACACCGGTTCTGCCGGTGGCCTGGCACCAACGTTGAAAGTGGGCGATATCGTTGTCTCGGACGAAGCACGTTATCA
CGACGCGGATGTCACGGCATTTGGTTATGAATACGGTCAGTTACCAGGCTGTCCGGCAGGCTTTAAAGCTGACGATAAACTGATCGCT
GCCGCTGAGGCCTGCATTGCCGAACTGAATCTTAACGCTGTACGTGGCCTGATTGTTAGCGGCGACGCTTTCATCAACGGTTCTGTTG
GTCTGGCGAAAATCCGCCACAACTTCCCACAGGCCATTGCTGTAGAGATGGAAGCGACGGCAATCGCCCATGTCTGCCACAATTTCAA
CGTCCCGTTTGTTGTCGTACGCGCCATCTCCGACGTGGCCGATCAACAGTCTCATCTTAGCTTCGATGAGTTCCTGGCTGTTGCCGCT
AAACAGTCCAGCCTGATGGTTGAGTCACTGGTGCAGAAACTTGCACATGGCTAA

ecluxS (His$_6$-tagged, *S*-ribosylhomocysteine lyase from *Escherichia coli*)

ATGGCTGCCGCGCGGCACCAGGCCGCTGCTGTGATGATGATGATGATGGCTGCTGCCCATATGCCGTTGTTAGATAGCTTCACAGTCG
ATCATACCCGGATGGAAGCGCCTGCAGTTCGGGTGGCGAAAACAATGAACACCCCGCATGGCGACGCAATCACCGTGTTCGATCTGCG
CTTCTGCGTGCCGAACAAAGAAGTGATGCCAGAAAGAGGGATCCATACCCTGGAGCACCTGTTTGCTGGTTTTATGCGTAACCATCTT
AACGGTAATGGTGTAGAGATTATCGATATCTCGCCAATGGGCTGCCGCACCGGTTTTTATATGAGTCTGATTGGTACGCCAGATGAGC
AGCGTGTTGCTGATGCCTGGAAAGCGGCAATGGAAGACGTGCTGAAAGTGCAGGATCAGAATCAGATCCCGGAACTGAACGTCTACCA
GTGTGGCACTTACCAGATGCACTCGTTGCAGGAAGCGCAGGATATTGCGCGTAGCATTCTGGAACGTGACGTACGCATCAACAGCAAC
GAAGAACTGGCACTGCCGAAAGAGAAGTTGCAGGAACTGCACATCTAG

ecmetf (His$_6$-tagged, methylene-H_4F reductase from *Escherichia coli*)

ATGGCTGCCGCGCGGCACCAGGCCGCTGCTGTGATGATGATGATGATGGCTGCTGCCCATATGAGCTTTTTTCACGCCAGCCAGCGGG
ATGCCCTGAATCAGAGCCTGGCAGAAGTCCAGGGGCAGATTAACGTTTCGTTCGAGTTTTTCCCGCCGCGTACCAGTGAAATGGAGCA
GACCCTGTGGAACTCCATCGATCGCCTTAGCAGCCTGAAACCGAAGTTTGTATCGGTGACCTATGGCGCGAACTCCGGCGAGCGCGAC
CGTACGCACAGCATTATTAAAGGCATTAAAGATCGCACTGGTCTGGAAGCGGCACCGCATCTTACTTGCATTGATGCGACGCCCGACG
AGCTGCGCACCATTGCACGCGACTACTGGAATAACGGTATTCGTCATATCGTGGCGCTGCGTGGCGATCTGCCGCCGGGAAGTGGTAA
GCCAGAAATGTATGCTTCTGACCTGGTGACGCTGTTAAAAGAAGTGGCAGATTTCGATATCTCCGTGGCGGCGTATCCGGAAGTTCAC
CCGGAAGCAAAAAGCGCTCAGGCGGATTTGCTTAATCTGAAACGCAAAGTGGATGCCGGAGCCAACCGCGCGATTACTCAGTTCTTCT
TCGATGTCGAAAGCTACCTGCGTTTTCGTGACCGCTGTGTATCGGCGGGCATTGATGTGGAAATTATTCCGGGAATTTTGCCGGTATC
TAACTTTAAACAGGCGAAGAAATTTGCCGATATGACCAACGTGCGTATTCCGGCGTGGATGGCGCAAATGTTCGACGGTCTGGATGAT
GATGCCGAAACCCGCAAACTGGTTGGCGCGAATATTGCCATGGATATGGTGAAGATTTTAAGCCGTGAAGGAGTGAAAGATTTCCACT
TCTATACGCTTAACCGTGCTGAAATGAGTTACGCGATTTGCCATACGCTGGGGGTTCGACCTGGTTTATAA

mmsahh (His$_6$-tagged)

ATGGCTGCCGCGCGGCACCAGGCCGCTGCTGTGATGATGATGATGATGGCTGCTGCCCATATGAGCGACAAACTGCCGTATAAAGTTG
CAGATATTGGTCTGGCAGCATGGGGTCGTAAAGCACTGGATATTGCAGAAAATGAAATGCCTGGTCTGATGCGTATGCGTGAAATGTA
TAGCGCAAGCAAACCGCTGAAAGGTGCACGTATTGCAGGTTGTCTGCACATGACCGTTGAAACCGCAGTTCTGATTGAAACCCTGGTT
GCACTGGGTGCAGAAGTTCGTTGGAGCAGCTGTAACATTTTTAGCACCCAGGATCATGCAGCAGCAGCAATTGCAAAAGCAGGTATTC
CGGTTTTTGCATGGAAAGGTGAAACCGATGAAGAATATCTGTGGTGTATTGAACAGACCCTGCACTTTAAAGATGGTCCGCTGAATAT
GATTCTGGATGATGGTGGTGATCTGACCAATCTGATTCATACCAAATATCCGCAGCTGCTGAGCGGTATTCGTGGTATTAGCGAAGAA
ACCACCACCGGTGTTCATAACCTGTATAAAATGATGAGCAACGGCATTCTGAAAGTTCCGGCAATTAATGTTAATGATAGCGTGACCA
AAAGCAAATTCGATAATCTGTATGGTTGTCGCGAAAGCCTGATTGATGGTATTAAACGTGCAACCGATGTTATGATTGCAGGTAAAGT
TGCCGTTGTTGCAGGTTATGGTGATGTTGGTAAAGGTTGTGCACAGGCACTGCGTGGTTTTGGTGCACGTGTGATTATTACCGAAATT
GATCCGATTAATGCACTGCAGGCAGCAATGGAAGGCTATGAAGTTACCACCATGGATGAAGCATGTAAAGAAGGCAACATTTTTGTTA
CAACCACCGGTTGCGTTGATATCATTCTGGGTCGTCATTTTGAGCAGATGAAAGATGATGCCATTGTGTGCAATATCGGCCATTTTGA
TGTTGAGATTGATGTGAAATGGCTGAATGAAAACGCCGTGGAAAAAGTGAACATTAAACCGCAGGTTGATCGCTATTGGCTGAAAAAT
GGTCGTCGTATTATTCTGCTGGCAGAAGGTCGTCTGGTTAATCTGGGTTGTGCAATGGGTCATCCGAGCTTTGTTATGAGCAATAGCT
TTACCAATCAGGTGATGGCACAGATTGAACTGTGGACCCATCCGGATAAATATCCGGTTGGTGTTCATTTCCTGCCGAAAAAACTGGA
TGAGGCAGTTGCAGAAGCACATCTGGGTAAACTGAATGTGAAACTGACCAAACTGACAGAAAAACAGGCACAGTATCTGGGTATGCCG
ATTAACGGTCCGTTTAAACCGGATCATTATCGCTATTAA
